# Dihydroorotate dehydrogenase regulates ferroptosis in neurons after spinal cord injury via the P53‐ALOX15 signaling pathway

**DOI:** 10.1111/cns.14150

**Published:** 2023-03-21

**Authors:** Dachuan Li, Xiao Lu, Guangyu Xu, Siyang Liu, Zhaoyang Gong, Feizhou Lu, Xinlei Xia, Jianyuan Jiang, Hongli Wang, Fei Zou, Xiaosheng Ma

**Affiliations:** ^1^ Department of Orthopedics, Huashan Hospital Fudan University Shanghai China

**Keywords:** ALOX15, DHODH, ferroptosis, P53, spinal cord injury

## Abstract

**Background:**

Spinal cord injury (SCI) is a highly disabling condition in spinal surgery that leads to neuronal damage and secondary inflammation. Ferroptosis is a non‐apoptotic type of cell death that has only recently been identified, which is marked primarily by iron‐dependent and lipid‐derived reactive oxygen species accumulation, and accompanied by morphological modifications such as mitochondrial atrophy and increase in membrane density. Dihydroorotate dehydrogenase (DHODH) is a powerful inhibitor of ferroptosis and has been demonstrated to inhibit cellular ferroptosis in tumor cells, but whether it can inhibit neuronal injury following spinal cord injury remains ambiguous.

**Methods:**

In this study, the effect of DHODH on neuronal ferroptosis was observed in vivo and in vitro using a rat spinal cord injury model and erastin‐induced PC12 cells, respectively. A combination of molecular and histological approaches was performed to assess ferroptosis and explore the possible mechanisms in vivo and in vitro.

**Results:**

First, we confirmed the existence of neuronal ferroptosis after spinal cord injury and that DHODH attenuates neuronal damage after spinal cord injury. Second, we showed molecular evidence that DHODH inhibits the activation of ferroptosis‐related molecules and reduces lipid peroxide production and mitochondrial damage, thereby reducing neuronal ferroptosis. Further analysis suggests that P53/ALOX15 may be one of the mechanisms regulated by DHODH. Importantly, we determined that DHODH inhibits ALOX15 expression by inhibiting P53.

**Conclusions:**

Our findings reveal a novel function for DHODH in neuronal ferroptosis after spinal cord injury, suggesting a unique therapeutic target to alleviate the disease process of spinal cord injury.

## Graphical abstract

After the occurrence of spinal cord injury, the neurons in the spinal cord will appear ferroptosis and further aggravate the spinal cord injury. Dihydroorotate dehydrogenase in mitochondria can inhibit the P53/ALOX15 signaling pathway, thereby inhibiting the lipid peroxidation and the ferroptosis of neurons, and finally alleviate the spinal cord injury.

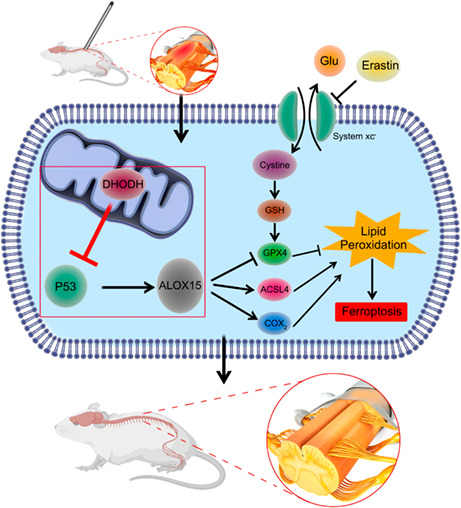

## 1 INTRODUCTION

Spinal cord injury (SCI) is a serious central neurotraumatic disease with high incidence of disability and mortality. Severe SCI can cause dysfunction below the level of injury, manifesting as paraplegia, loss of sensation, and other spinal cord abnormalities, which results in heavy financial strain on individuals, families, and society. The complex and diverse pathophysiological processes and microenvironmental imbalances following SCI, including excitotoxicity, lipid peroxidation, neuroinflammation, ischemia‐reperfusion injury, and the production of free radicals,^1^, ^2^ are the important reasons for the difficult repair of SCI. There are still no effective repair options available. In order to enhance the prognosis of individuals with spinal cord injury, more in‐depth mechanisms need to be researched.

The iron‐dependent form of programmed death known as ferroptosis was first identified in 2012.^3^ Its notable characteristics include the accumulation of lipid reactive oxygen species, accumulation of iron ions, and lipid peroxidation. The main mechanism and signaling pathway of ferroptosis are complex and closely related to cystine/glutamate reverse transporter, glutathione peroxidase 4, ferroptosis inhibitory protein 1, and dihydroorotic acid dehydrogenase. Its important role in central neurodegeneration and traumatic injury is increasingly being discovered as one of the important modes of neuronal cell death.^4^ Morphological, biochemical, and molecular evidence for the presence of ferroptosis in SCI pathology has been found. The fragmentation and hemolysis of erythrocytes following spinal cord injury release large amounts of free iron, resulting in local iron overload^5^; at the same time, the stress response to injury activates large amounts of lipid reactive oxygen species, leading to the accumulation of lipid reactive oxygen species,^6^ which is further increased by the iron overload, revealing the involvement of ferroptosis in secondary damage to spinal cord tissue after SCI.

The inner membrane of mitochondria contains an enzyme called dihydroorotate dehydrogenase (DHODH), which is flavin‐dependent. DHODH is crucial for the de novo synthesis of pyrimidines. It catalyzes the fourth step in the synthesis of pyrimidine nucleotides (oxidation of DHO to OA and reduction of ubiquinone to CoQH_2_).^7^ A recent study by Mao et al.^8^ showed that DHODH has been demonstrated to prevent mitochondrial ferroptosis by regulating CoQH2 synthesis in the inner mitochondrial membrane. DHODH is a key mechanism that inhibits the anticancer activity of ferroptosis. According to their research, two important metabolic enzymes that detoxify lipid peroxides which accumulate in mitochondria are DHODH and mitochondrial GPX4. Therefore, DHODH and GPX4 inhibit ferroptosis by preventing lipid peroxidation in mitochondria, potentially offering a promising method for reducing ferroptosis in neurons following spinal cord injury.

In recent years, the involvement of P53 in ferroptosis has gradually become an emerging research area. Studies have revealed that the P53's ability to regulate ferroptosis contributes to the tumor suppressive function of P53, and that the accumulation of MUT P53 protein in cancer cells makes cancer cells sensitive to ferroptosis. Also, in clinical studies related to autoimmune diseases, cancer, and viral infections, it has been demonstrated that intracellular DHODH has a regulatory role on P53.^9^, ^10^ In addition, it has been found that levels of ALOX15 (arachidonic acid 15‐lipoxygenase), a member of the lipoxygenase family that oxidizes PUFA and causes ferroptosis as a result of oxidative stress, are increased following P53 induction and that ROS‐induced ferroptosis can be effectively blocked by the ALOX15‐specific inhibitor PD146176.^11^, ^12^ In addition, the results of the study by et al. showed that increased expression of ALOX15 sensitized cells to ferroptosis.^13^, ^14^ This suggests that ALOX15 is a mediator of P53‐induced ferroptosis. However, the association between P53/ALOX15 and ferroptosis in spinal cord injury remains unclear, and further study needs to be done because of its potential molecular mechanism.

To date, no studies have been reported on the inhibition of ferroptosis by DHODH in spinal cord injury. Therefore, we constructed animal as well as neuronal models of ferroptosis in rats and PC12 cells in order to study the role of DHODH. In addition, experiments were carried out to confirm that DHODH can inhibit ferroptosis of neurons in spinal cord injury through P53/ALOX15 pathway, promote neuronal survival, and alleviate spinal cord injury.

## 2 MATERIALS AND METHODS

### 2.1 Animals

Forty female Sprague–Dawley rats (200–300 g, 8 weeks old) were purchased from the Animal Experiment Center of Fudan University. To exclude gender differences, all animals were female rats. The rats were raised in standard cages (4 rats/cage) in a pathogen‐free facility with a 12‐h light/dark cycle and controlled temperature and humidity, and were allowed to acclimatize for 1 week prior to the experiments. All experiments were conducted in accordance with the experimental protocol approved by the Animal Protection and Utilization Committee of Fudan University (No. 202203013S).

### 2.2 Spinal cord injury and drugs treatment

Forty rats were randomly divided into four groups: sham operation group (*n* = 10), SCI group (*n* = 10), SCI + ferroptosis inhibitor group (SCI + ferrostatin‐1) (*n* = 10), and SCI + DHODH Inhibitor group (SCI + teriflunomide) (*n* = 10). Ten rats in the sham group only received laminectomy without SCI. To induce spinal cord injury, spinal cord injury surgery was performed in the middle thoracic region of rats (T8–T9).^15^ In short, animals were anesthetized by intraperitoneal injection with a mixture of 70 mg kg^−1^ ketamine (Hengrui)^16^ and 5 mg kg^−1^ toluene thiazide (McLean). Each rat was placed in a prone position on the operating table with full exposure of the spine, followed by laminectomy of the T8/T9 lamina. A customized impactor (2 mm in diameter, 12.5 g in weight, and 1 cm in height) was utilized to strike the totally exposed spinal cord at T8/T9, resulting in a spinal cord contusion. The same surgery was carried out without injuring the spinal cord in the sham group, and the skin was sutured following hemostasis. The SCI + ferroptosis inhibitor group and SCI + DHODH inhibitor group were injected with NanoFil microinjection system after surgery referring to the dose and injection method of Ge and Rzagalinski et al.^17^, ^18^ Ferrostatin‐1 (0.7 mg kg^−1^ in DMSO, MCE) and teriflunomide (10 mg kg^−1^ in DMSO, MCE) 10 μL each have been slowly microinjected into the spinal cord injury at a depth of about 1 mm. The Sham group and SCI group were injected with the same volume of DMSO. Throughout the experiment, the rats were observed daily for health, cage activity, and infection. The antibiotic gentamicin sulfate (4 mg kg^−1^; Huachu) and meloxicam (1 mg kg^−1^; Boehringer) were administered to the rats for 3 days during their postoperative recovery. Up until the rats started urinating on their own again, manual bladder massage was performed twice daily.

### 2.3 Magnetic resonance imaging of spinal cord

Four weeks after SCI, anesthetized rats were subjected to MR imaging by intraperitoneal injection (3.0T, Prism; Siemens). After anesthesia was effectively achieved, the rats were placed on an MRI coil. The spine was adjusted to go straight and straight through the coil. MRI images were evaluated by three orthopedic researchers.

### 2.4 Evaluation of the functional recovery of rats with SCI

The motor recovery of the rats in each group was assessed according to the Basso, Beattie, and Bresnahan (BBB) motor score.^19^, ^20^ The scale is based on allowing the rats to walk freely for 5 min in a 90 cm field while closely observing the movement of the hind limbs as well as coordination. The Sham group, SCI group, SCI + ferroptosis inhibitor group, and SCI + DHODH inhibitor group were assessed on days 1, 3, 7, 14, 21, and 28 postoperatively. The experimental conditions of the rats were not known to the assessors and the assessments were repeated three times and recorded immediately. Scores ranged from 0 to 21, with 0 being no motor function and 21 being normal. Footprints were recorded to analyze the ability of the limbs to coordinate their movements by coloring the hind limbs with red ink. Next, place the mice on white A2 paper (594 × 420 mm) and make them walk freely.

### 2.5 Flexibility of the ankle joint

**FIGURE 1:**
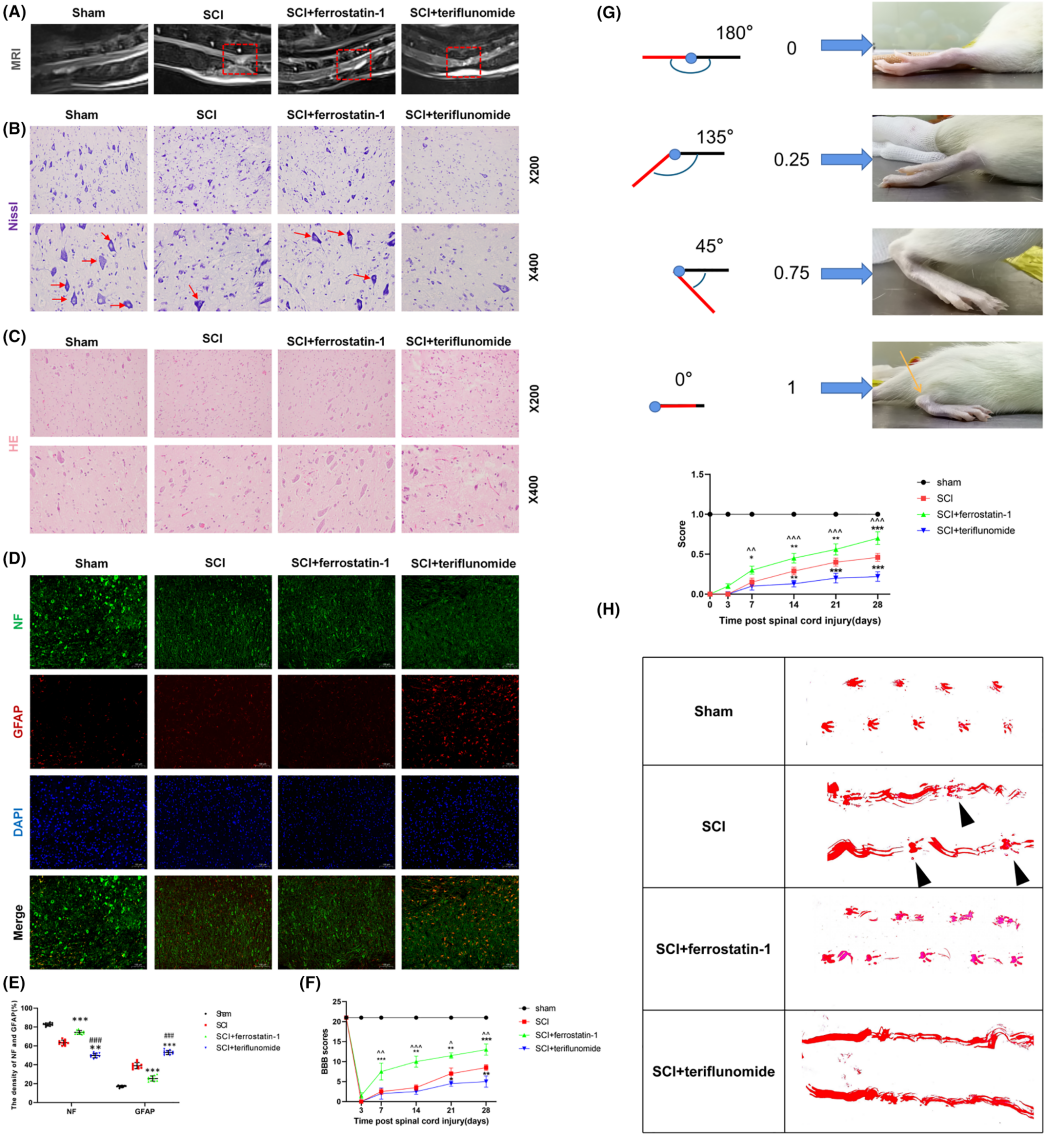
Dihydroorotate dehydrogenase can improve tissue damage of neurons after spinal cord injury and promote functional recovery. (A) Representative MR images of the spinal cord of rats in each group at 4 weeks after spinal cord injury, with injury areas marked by red boxes. (B) Nissl staining and Nissl staining for partial enlarged images. The number of motor neurons in the anterior horn of spinal cord was observed and analyzed by Nissl staining. The arrow represents neurons containing Nissl bodies (*n* = 10 for each group, scale bar = 20 μm). (C) HE staining images of representative spinal cord cross‐sections of each group (*n* = 10 for each group, scale bar = 50 μm). (D, E) NF (Green) and GFAP (Red) immunofluorescence staining. Each group reflects the functional status of nerve fibers, the ability to maintain continuous nerve fibers and inhibit astrocyte proliferation. (Scale bar: 100 μm. Image J software is used to measure the density of GFAP and NF in three randomly selected fields of vision.) (F) BBB scores were used to assess changes in hind limb motor recovery at 3, 7, 14, 21, and 28 days after spinal cord injury (*n* = 10 for each group). (G) Schematic score representation of the evaluation of ankle flexibility and the ankle flexibility score of each group (*n* = 10 for each group). (H) Representative images of footprints from each group. The hindlimbs were displayed in red (*n* = 10 for each group). (All the data are expressed as means ± SD, one or two‐way ANOVA followed by Tukey's post hoc test was applied; *p < 0.05, **p < 0.01, ***p < 0.001 vs. SCI; ^#^ *p* < 0.05, ^##^ *p* < 0.01, ^###^ *p* < 0.001 vs. SCI + ferrostatin‐1; ^^^ *p* < 0.05, ^^^^ *p* < 0.01, ^^^^^ *p* < 0.001 vs. SCI + teriflunomide.)

The spasticity of the muscles involved in the ankle dorsiflexors and plantar flexors was evaluated from the postoperative analysis of rats,^21^ and the time of evaluation was consistent with BBB. The rats were also scored according to different flexion states of the hind limbs, with reference to Dolci et al.^22^ ‘0’ for lack of movement (spasticity, corresponding to a 180° angle between the tibialis anterior muscle and the paw), ‘0.25’ for 135° angle, ‘0.75’ to 45° angle, and ‘1’ to normal movement corresponding to a 0° angle (Figure 1G).

### 2.6 Cell culture

PC12 belongs to the adrenal pheochromocytoma cell line, which is neurocrist‐derived and has nerve cell characteristics, and is commonly used in the study of nerve development and function. As PC12 cells have physiological properties similar to neurons, PC12 is also an excellent in vitro cell model for many scholars to study the pathogenesis and progression of various neurological diseases (such as neurotoxicity and neuroinflammation).^23^ The pc12 cells were purchased from iCell, China and cultured in DMEM (Gibco) containing 10% fetal bovine serum, 50 g/mL streptomycin (Invitrogen) and 50 U/mL penicillin at 37°C and 5% CO_2_. Ferroptosis of neurons was simulated. OE‐DHODH, OE‐P53, and OE‐ALOX15 plasmids were constructed by Genechem Ltd., and according to the manufacturer's instructions, Lipofectamine 2000 reagent (Invitrogen) was used to transfect PC12 cells. Ferroptosis agonist (Erastin) (5 μM) and ferrostatin‐1 (1 μM) were added into the plasmids 24 h after formation. The cells were treated for 24 h before subsequent analysis.

### 2.7 Quantitative real‐time polymerase chain reaction

RNA was extracted from harvested spinal cord tissues and PC12 cells using TRIpure reagent (EP013; ELK Biotechnology) according to the manufacturer's instructions. qPCR was performed with the use of QuFast SYBR Green PCR system (Life Technologies). Relative mRNA expression was calculated by the comparative ΔΔCT method using GAPDH as the housekeeping gene. The primers used in this study are shown in Table S1.

### 2.8 Western blot

In a solution containing a protease inhibitor (AS1008; Aspen), PC12 cells were dissolved on ice for 30 min. BCA protein assay kit was used to measure the protein concentration (PC0020; Solarbio). The protein samples were heated at 100°C for 10 min to completely denature the proteins. The protein samples were then separated by SDS‐PAGE electrophoresis and transferred to PVDF membranes (IPVH00010; Millipore). After being blocked for an hour with 5 percent skim milk, the membrane was incubated with the primary antibody at 4°C overnight. The antibodies used in this study consisted of anti‐DHODH (1:1000, 14877‐1‐AP; Proteintech), anti‐ACSL4 (1:1000, 22401‐1‐AP; Proteintech), anti‐COX2 (1:1000, 66351‐1‐Ig; Proteintech), anti‐GPX4 (1:1000, 67763‐1‐Ig; Proteintech), anti‐ALOX15 (1:1000, ab244205; Abcam), anti‐P53 (1:1000, AB_297667; Abcam). After being washed three times with TBST, the membrane was incubated for 10 min at room temperature with a secondary antibody that had been enzyme‐labeled. Imaging was carried out using an ECL chemiluminescence substrate (BL520B; Biosharp). Band intensities were quantified using Image J software.

### 2.9 Immunofluorescence assessment

Spinal cord tissue samples were collected as described above. After cell culture treatment, PC12 cell samples were fixed with 4% paraformaldehyde for 20 min and washed with PBS for three times, 5 min each. Soak in 0.5% Triton X‐100 for 20 min and block with 1% bovine serum albumin for 60 min. Incubate overnight with primary antibody at 4°C. Then PBS was washed for three times and the corresponding secondary antibody was incubated at 37°C for 40 min. All images were captured with the Nikon ECLIPSE CI‐L microscope (Nikon). The fluorescence intensity was quantified by Image J software.

### 2.10 Cell viability assays

Cell counting kit‐8 (CCK‐8) (C0038; Beyotime) was used to assess cell viability in accordance with the manufacturer's recommendations. Simply said, PC12 cells were seeded onto 96‐well plates at a rate of 10,000 cells per well and cultured for 24 h at 37°C in a CO_2_ (5%) incubator. The medium of each group was changed to 100 μL cell sample corresponding medium containing certain concentrations of drugs, and the control group was changed to medium containing solvent. Then, add 10 μL CCK‐8 solution to all the wells and incubate for 2 h at 37°C. Finally, OD values were measured at 450 nm with a microplate meter (DR‐200BS; Diatek), and relative cell viability was expressed as a control percentage.

### 2.11 Measurement of malondialdehyde

Malondialdehyde (MDA) content assay kit (A003‐1; Nanjing Jiancheng Biotechnology Co., Ltd.) cellular MDA content was detected according to thiobarbituric acid (TBA) reactive assay of cellular malondialdehyde content. Samples were first collected and then operated according to the kit instructions to calculate the MDA content of each group of samples.

### 2.12 Iron concentration determination

The collection of samples was first performed, and the spinal cord iron levels of rats in each group were determined using a tissue iron assay kit (Nanjing Jiancheng Biotechnology Co., Ltd.) according to the manufacturer's instructions. To determine the iron concentration, measure and read the optical density (OD) value at 520 nm using a spectrophotometer (Varioskan Flash; Thermo Scientific), then determine the iron concentration in all samples by contrasting the samples' OD with the standard curve.

### 2.13 Histological analysis and neural‐like cell assessments

The rats were deeply anesthetized with ketamine (0.1 mg g^−1^) 6 weeks after spinal cord injury. Then, after they were executed, spinal cord tissue fixed with 4% (w/v) paraformaldehyde for 24 h was used to obtain frozen sections of 20 μm thickness using a cryo‐tissue slicer. The tissues underwent a 48‐h fixation period, a 4‐h rinse under running water, gradient dehydration, an overnight soak in xylene, and paraffin embedding. Finally, a paraffin slicer produced a paraffin segment with a thickness of 7 m. After being submerged in water for 5 min, paraffin slices were stained for 2 min with eosin and 5 min with hematoxylin (both from Solarbio). The characteristics of cells and extracellular matrix were observed. Light microscopy was used to observe changes in the number of Nisin bodies within the anterior horn of the spinal cord in sections. In addition, neuronal cell numbers and functional status were reflected by neurofilament protein (NF) staining, and glial fibrillary acidic protein (GFAP) staining.

### 2.14 Transmission electron microscopy

The PC12 cells were fixed in 2% glutaraldehyde for 2 h and then transferred to 1% citric acid for fixation. They were then soaked in uranyl acetate solution and dehydrated with gradient acetone. The samples were then embedded in epoxy resin and sectioned at 70–90 nm. They were then placed on a copper trough grid, re‐stained with lead citrate, and the ultrastructure of the mitochondria was observed by transmission electron microscopy (TEM).

### 2.15 Measurement of mitochondrial membrane potential (Δψm) using flow cytometry

The mitochondrial membrane potential was measured using JC‐1. The pc12 cells were collected enumerated, and then resuspended at a concentration of 10^5^ cells/mL in frozen PBS. A crucial metric for measuring mitochondrial integrity, Δψm, was evaluated using JC‐1 fluorescent probes from Beyotime Biotech in Nanjing, China. JC‐1 was diluted 1:500 in medium without serum to a final concentration of 10 μg/mL. After being incubated for 20 min at 37°C in a cell culture incubator, the cells were mixed upside down every 3–5 min to ensure that the probe made complete contact with the cells. They were then rinsed three times with a culture medium devoid of serum to remove any remaining probe. Following staining as directed by the manufacturer, analysis is conducted using fluorescence‐activated cell sorting (FACS).

### 2.16 Statistical analysis

For all in‐vitro experiments, *n* = 3 are biological replicates, and the experiments were carried out in at least two independent experiments. SPSS 22.0 (IBM) statistical software was used for analysis. The measurement data are expressed as the means ± standard deviation. When the normal distribution was satisfied (Shapiro–Wilk *W* test) and the variance was homogeneous, the data between the two groups were compared by *t*‐test, and the data between the multiple groups were compared by single factor analysis of variance (ANOVA). When the data does not follow the normal distribution or the variance is uneven, the data between the two groups were compared by Wilcoxon rank‐sum test and the data between the multiple groups were compared by Kruskal–Wallis test. The LSD test was used for pairwise comparison. The bilateral inspection level was *α* = 0.05. A *p* value of less than 0.05 was considered to be statistically significant. The figures were drawn by Figdraw and GraphPad Prism 9 (GraphPad).

## 3 RESULTS

### 3.1 DHODH can improve tissue damage of neurons after spinal cord injury and promote functional recovery

Four weeks after spinal cord injury, SCI + teriflunomide group showed finer sites of spinal cord injury on MR imaging, more soft tissue swelling, and more hypersignal areas compared with other groups, indicating more severe severity of spinal cord injury and inflammation after DHODH inhibition (Figure 1A, Figure S1). According to Nissl staining, the SCI + teriflunomide group had the lowest number of Nissl bodies compared to the other three groups (Figure 1B). HE staining was also used for pathological examination to observe gross morphology. HE staining showed that inflammatory response and tissue damage of SCI + teriflunomide group were significantly heavier than other groups after spinal cord injury. In this group, inflammatory cells and tissue edema increased (Figure 1C). In addition, further immunofluorescence staining of nerve cells showed that at the site of spinal cord injury, the expression of NF and GFAP in spinal cord neurons treated with DHODH inhibitor was significantly disordered and the density of NF‐positive cells was significantly reduced compared with SCI group and SCI + ferrostatin‐1 group, while the number of GFAP‐positive cells was higher than that of other groups (Figure 1D,E). The effect of DHODH on motor recovery of rats was detected, and BBB scores were measured at 3, 7, 14, 21, and 28 days after spinal cord injury. All rats had a BBB score of 21 before spinal cord injury and an immediate score of 0 after spinal cord injury, indicating that acute spinal cord injury resulted in lower limb paralysis. Scores began to increase over time, but were still lower in each group than in the sham group (Figure 1F). In the first week after the spinal cord injury, SCI + ferrostatin‐1 group recovered the fastest, and then the recovery rate gradually slowed down. SCI + teriflunomide group had slower recovery of motor function compared with other groups and showed a significant difference (Figure 1F). At the last assessment, almost all rats in the SCI + ferrostatin‐1 group were able to stand on both legs and move almost smoothly. Muscle spasm is a typical sign of upper motor neuron injury, a common feature of spinal cord injury, and likewise a major cause of disability in individuals with various central nervous system disorders and trauma.^24^ Through the assessment of the ankle joint of the rats (Figure 3G and Section 2.5), it was found that the spasticity of the hind limbs of the rats gradually recovered with the progress of time, and the recovery of SCI + ferrostatin‐1 group was the best, while the recovery of SCI + teriflunomide group was the worst after the suppression of DHODH, which was almost in the spasticity paralysis state. Then we examined whether DHODH deficiency after spinal cord injury affects motor function. As shown in Figure 3H, footprint behavior analysis revealed that severe imprinting disorder with waveform and dragging motion was observed after SCI in rats added with DHODH inhibitor. Although the rats in the spinal cord injury group had the same footprint disorder, a few off‐ground movements of hind limbs (black triangle) were clearly visible. The SCI + ferrostatin‐1 group had the most coherent and did not show distinctive signs of shuffling. Therefore, these results suggest that inhibition of DHODH significantly delays recovery of motor function after spinal cord injury and aggravates neuronal injury. In other words, DHODH improves tissue damage of neurons after spinal cord injury.

### 3.2 DHODH can inhibit neuronal ferroptosis after spinal cord injury and relieve spinal cord injury

**FIGURE 2:**
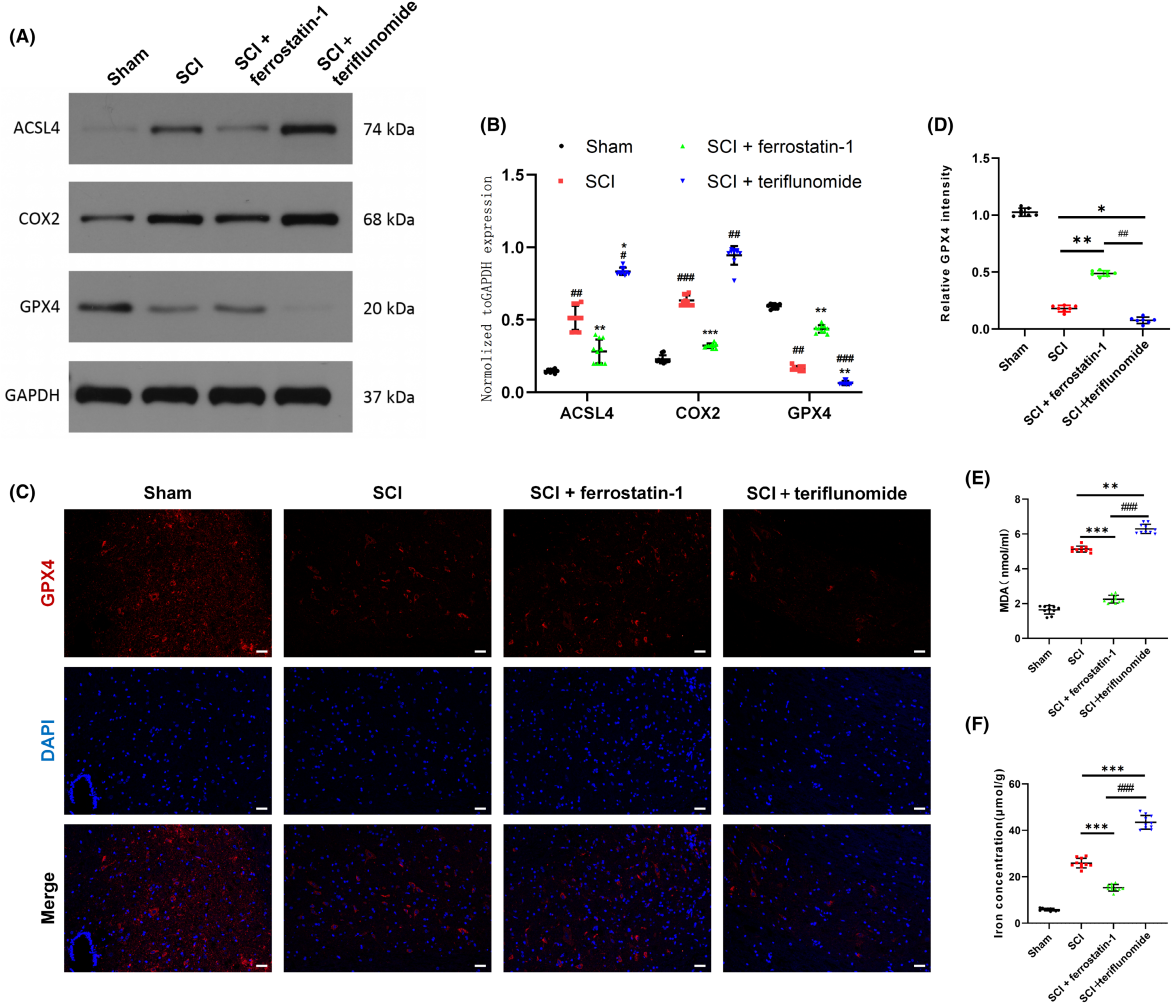
Dihydroorotate dehydrogenase can inhibit neuronal ferroptosis after spinal cord injury and relieve spinal cord injury. (A) Expression of ferroptosis‐related molecules ACSL4, COX2 and GPX4 were detected by Western blot. (B) Quantification of ACSL4, COX2 and GPX4 expressions (*n* = 10 for each group). (C) The expression level of GPX4 in each group was detected by immunofluorescence staining (*n* = 10 for each group, scale bar = 50 μm). (D) Quantitative statistical analysis of GPX4 immunofluorescence staining in each group (*n* = 10 for each group, scale bar = 50 μm, relative content of GPX4 was calculated by Image J software). (E) MDA content and analysis in each group (*n* = 10 for each group). (F) Iron concentration at the injury site after spinal cord injury in each group (*n* = 10 for each group). (All the data are expressed as means ± SD, one or two‐way ANOVA followed by Tukey's post hoc test was applied. *p < 0.05, **p < 0.01, ***p < 0.001 vs. SCI; ^#^ *p* < 0.05, ^##^ *p* < 0.01, ^###^ *p* < 0.001 vs. SCI + ferrostatin‐1.)

Western Blot results showed that ACSL4 and COX2 expression were significantly increased in SCI + teriflunomide group compared with the other three groups; compared with SCI group, ACSL4 and COX2 expression were decreased in SCI + ferrostatin‐1 group (Figure 2A,B). In addition, the expression level of GPX4 in the SCI + teriflunomide group was significantly lower than that in the other groups (Figure 2A,B). GPX4, ACSL4, and COX‐2 are widely regarded as important regulators of ferroptosis. These results suggest that ferroptosis is involved in spinal cord injury. The neuronal ferroptosis was more severe after DHODH inhibition. Second, immunofluorescence also showed that the expression of GPX4 in spinal cord neurons of the other three groups was significantly increased compared with SCI + teriflunomide‐treated spinal cord neurons (Figure 2C,D). The MDA test results also demonstrated the presence of oxidative stress‐induced lipid peroxidation after spinal cord injury, with higher levels of MDA in the SCI + teriflunomide group compared with the results of the other groups (Figure 2E). Iron was the main trigger for ferroptosis, and we subsequently identified Iron concentration. The results showed that iron concentration was significantly increased in the spinal cord after SCI, and it was significantly decreased by ferrostatin‐1 and in the SCI + teriflunomide group its level was significantly higher than that in the other groups (Figure 2F). The results of this part of the in vivo study indicate that neuronal ferroptosis occurs after spinal cord injury. At the same time, due to the decreased expression of DHODH, the expression of ferroptosis‐promoting molecules ACSL4 and COX2 is upregulated, and the expression of ferroptosis‐inhibiting molecule GPX4 is downregulated, and then the detection of MDA and iron concentration further indicated that DHODH inhibited the ferroptosis of neurons after SCI, and played a protective effect on neurons.

### 3.3 DHODH can inhibit ferroptosis of neurons in vitro after spinal cord injury

**FIGURE 3:**
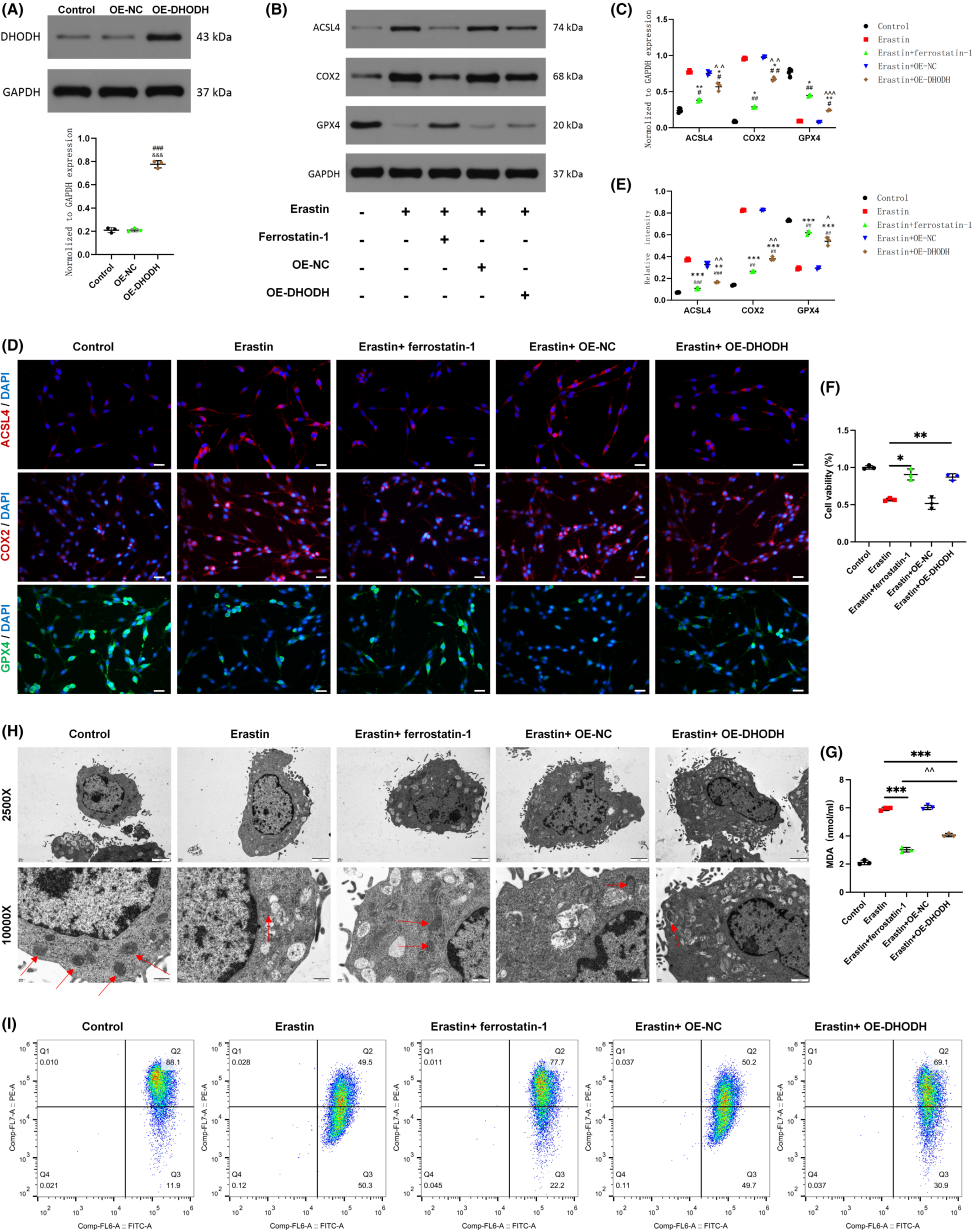
Dihydroorotate dehydrogenase can inhibit ferroptosis of neurons in vitro after spinal cord injury. PC12 cells were treated with erastin (0.5 μM) or ferrostatin‐1 (1 μM) for 24 h. (A) Expression of OE‐DHODH in transfected pc12 cells. (B, C) Western blot detection and statistical analysis of ASCL4, COX2, GPX4 expression in PC12 cells in each group. (D, E) Statistical analysis of the expression level and quantification of GPX4 in each group by immunofluorescence staining (scale bar = 50 μm, the relative content of GPX4 was calculated by Image J software). (F) The CCK‐8 detection kit was used to detect the survival of PC12 cells in each group in the absence or presence of erastin. (G) The content and analysis of MDA in each group after treatment. (H, I) Transmission electron microscopy representative images of mitochondrial ultrastructure in PC‐12 cells after treatment with erastin (scale bar = 2 μm, scale bar = 500 nm, red arrows indicate mitochondria), JC‐1 was used to detect mitochondrial membrane potential in pc12 cells. (All the data are expressed as means ± SD, *n* = 3, one or two‐way ANOVA followed by Tukey's post hoc test was applied *p < 0.05, **p < 0.01, ***p < 0.001 vs. Erastin; ^#^ *p* < 0.05, ^##^ *p* < 0.01, ^###^ *p* < 0.001 vs. Control; ^^^ *p* < 0.05, ^^^^ *p* < 0.01, ^^^^^ *p* < 0.001 vs. Erastin + ferrostatin‐1; ^&^ *p* < 0.05, ^&&^ *p* < 0.01, ^&&&^ *p* < 0.001 vs. OE‐NC.)

To determine whether DHODH also has the ability to inhibit ferroptosis in vitro, we used PC12 to detect the level of ferroptosis in cells and constructed PC12 cells with upregulation of DHODH expression. It was treated with the ferroptosis agonist erastin. The results of quantitative real‐time polymerase chain reaction (qRT‐PCR) and WB showed that the efficiency of DHODH overexpression met the experimental requirements (Figure 3A, Figure S3A). In the current experiment, the overall levels of ACSL4, COX2 increased with the addition of erastin, and the expression of GPX4 decreased and both were reversed with the upregulation of DHODH and the addition of ferrostatin‐1, but the reversal effect seemed to be reversed after the addition of ferrostain‐1 more significantly (Figure 3B,C). Immunofluorescence showed that the expression level of GPX4 changed similarly to the upregulation of DHODH (Figure 3D,E). The results of CCK‐8 assay showed that DHODH had no significant cytotoxic effect on PC12 cells. Furthermore, DHODH and ferrostatin‐1 significantly reversed erastin‐induced loss of PC12 cell viability (Figure 3F). In order to further clarify the protective mechanism of DHODH on PC12 cells, whether it can inhibit the lipid peroxidation of PC12 cells. After erastin treatment, the MDA levels of each group were tested, and DHODH significantly decreased the intracellular MDA level, but the erastin +ferrostain‐1 group seemed to decrease more (Figure 3G). Since mitochondrial changes are considered to be the main morphological feature that distinguishes them from other types of cell death,^25^ DHODH is also located in mitochondria, and we used electron microscopy to observe the situation of mitochondria in cells. The results showed that the mitochondria of PC12 cells treated with erastin had atrophied, which the shape was irregular, and the mitochondrial cristae were reduced or completely disappeared, and the mitochondrial membrane collapsed, which was considered to be the obvious ferroptosis of the cells. The mitochondrial morphology was significantly improved after DHODH treatment, with regular morphology and less vacuolation (Figure 3H). Furthermore, we used the JC‐1 probe to perform mitochondrial membrane potential detection and found that red fluorescence was decreased in pc12 cells treated with erastin, while green fluorescence was increased, consistent with mitochondrial damage. The mitochondrial membrane potential in the upregulated DHODH expression group was significantly higher than that in the erastin group (Figure 3I). The results of the ferrostatin‐1 addition group were similar to those of the DHODH overexpression group. These results together suggest that DHODH can inhibit neuronal ferroptosis in vitro.

### 3.4 DHODH can alleviate neuronal ferroptosis by regulating P53

**FIGURE 4:**
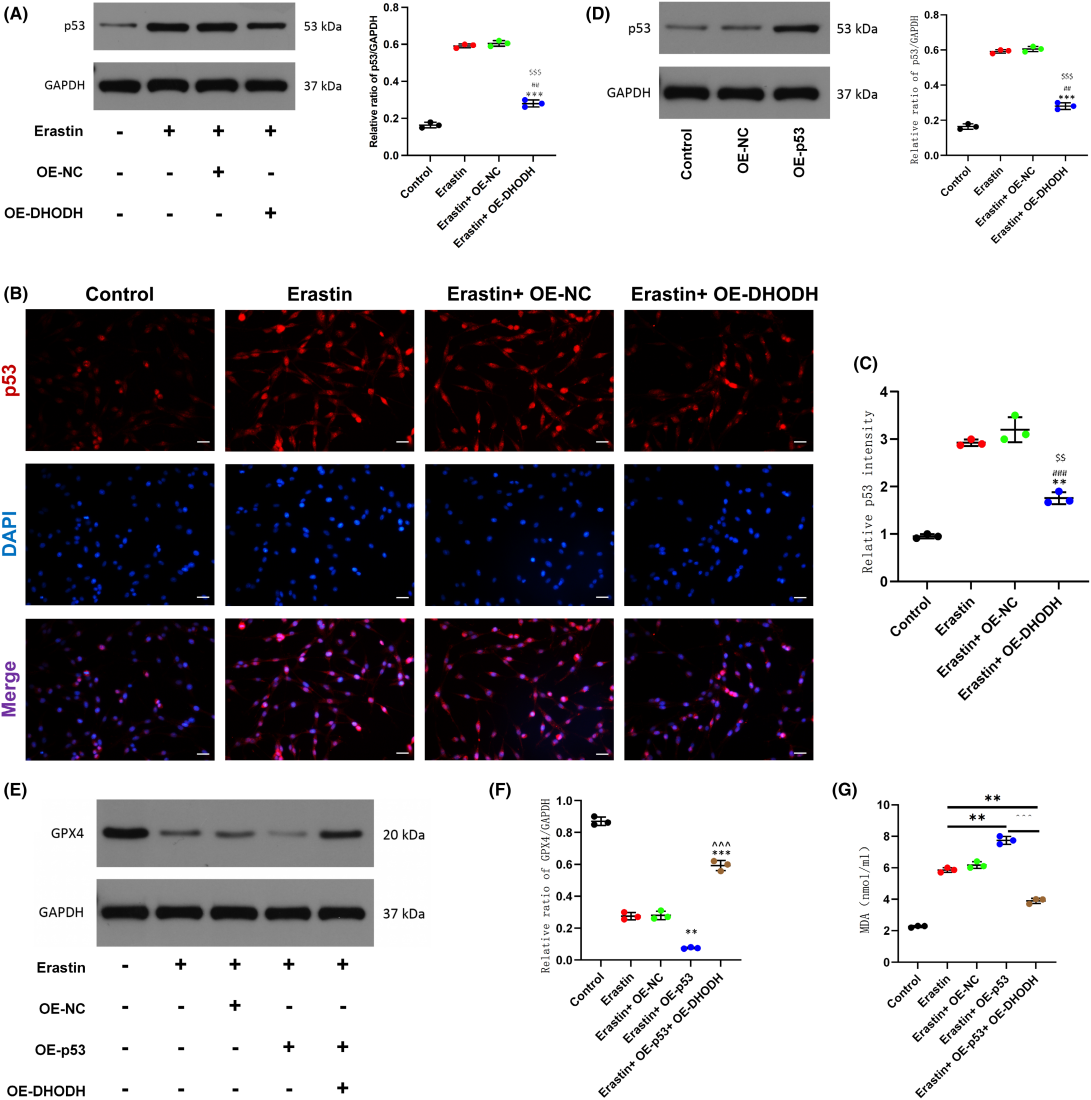
Dihydroorotate dehydrogenase can alleviate neuronal ferroptosis by regulating P53. pc12 cells were treated with erastin (0.5 μM) for 24 h. (A) Western blot detection of P53 expression and quantitative statistical analysis. (B, C) Immunofluorescence staining to detect P53 expression and statistical analysis in each group (scale bar = 50 μm, the relative content of GPX4 was calculated by Image J software). (D) Expression of OE‐P53 in transfected pc12 cells. (E, F) Western blot detection of GPX4 expression after up‐regulation of P53 or (and) up‐regulation of DHODH and quantitative statistical analysis. (G) MDA assay to detect the release of MDA in cells of different treatment groups. (All the data are expressed as means ± SD, *n* = 3 one‐way ANOVA followed by Tukey's post hoc test was applied *p < 0.05, **p < 0.01, ***p < 0.001 vs. Erastin; ^#^ *p* < 0.05, ^##^ *p* < 0.01, ^###^ *p* < 0.001 vs. Control; ^^^ *p* < 0.05, ^^^^ *p* < 0.01, ^^^^^ *p* < 0.001 vs. Erastin + OE‐P53; ^&^ *p* < 0.05, ^&&^ *p* < 0.01, ^&&&^ *p* < 0.001 vs. OE‐NC; ^$^ *p* < 0.05, ^$$^ *p* < 0.01, ^$$$^ *p* < 0.001 vs. Erastin + OE‐NC.)

In order to further study the mechanism of DHODH regulating neuronal ferroptosis, previous studies have shown that there may be a regulatory relationship between DHODH and P53 in other tissues and cells.^26^ By upregulating the expression of DHODH, we used Western blot and immunofluorescence staining to detect cells expression of P53. As shown in Figure 4A–C, the expression and the fluorescence intensity of P53 were significantly decreased in pc12 cells overexpressing DHODH, indicating that DHODH downregulated the expression of P53. In addition, we observed that DHODH regulates ferroptosis through P53 by synergistically upregulating the expression of DHODH and P53 in erastin‐treated pc12 cells. The results of qRT‐PCR and WB showed that the efficiency of P53 overexpression met the experimental requirements (Figure 4D, Figure S3B). Western blot compared the expression of GPX4 in each group. The results showed that after upregulation of P53, the expression of GPX4 was significantly decreased compared with the erastin group, and the expression was reversed after synergistic upregulation of DHODH (Figure 4E,F). MDA detection was used to further confirm the role of P53 and DHODH in regulating ferroptosis. Upregulation of P53 increased intracellular MDA content, while upregulation of DHODH reversed this effect (Figure 4G). To sum up, these results collectively suggest that DHODH regulates neuronal ferroptosis by inhibiting the expression of P53.

### 3.5 ALOX15 is located downstream of DHODH and P53, and its expression is regulated by both, through in vitro and in vivo experiments

**FIGURE 5:**
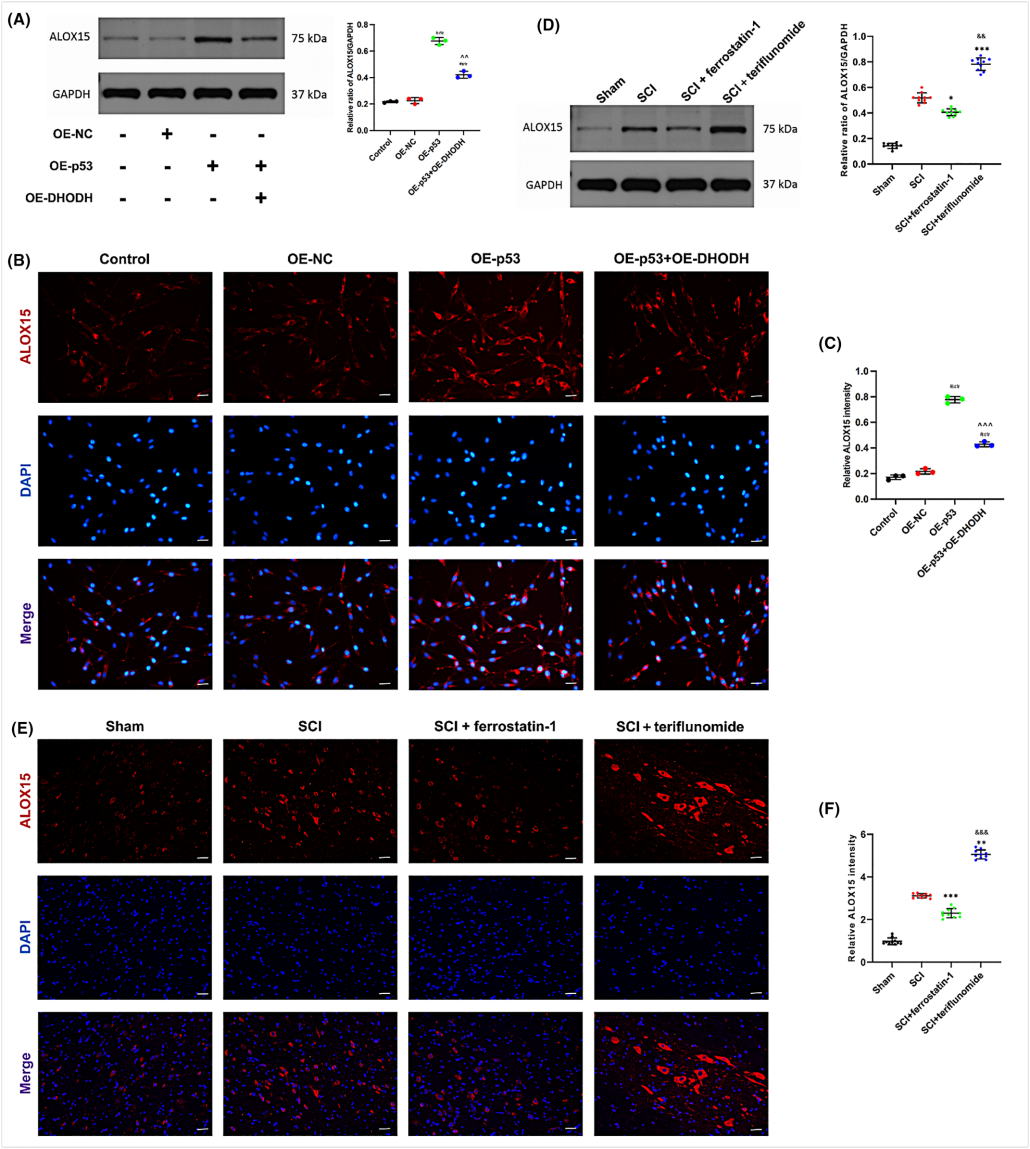
ALOX15 is located downstream of DHODH and P53, and its expression is regulated by both, through in vitro and in vivo experiments. (A–C) ALOX15 expression and quantitative statistical analysis after up‐regulation of DHODH or (and) P53 was detected by Western blot in pc12 cells (*n* = 3). The expression of ALOX15 in the cells of each group was evaluated by immunofluorescence staining (*n* = 3, scale bar = 50 μm, the relative content of ALOX15 was calculated by Image J software). (D–F) In rats with spinal cord injury, western blot was used to detect and quantify the expression of ALOX15 in each group. Inhibition of DHODH up‐regulated ALOX15 expression (*n* = 10 for each group). The immunofluorescence staining of spinal cord injury rats was used to detect the immunofluorescence intensity of each group (*n* = 10 for each group, scale bar = 50 μm, relative content of ALOX15 was calculated by Image J software). (All the data are expressed as means ± SD, one‐way ANOVA followed by Tukey's post hoc test was applied. ^#^ *p* < 0.05, ^##^ *p* < 0.01, ^###^ *p* < 0.001 vs. Control; ^^^ *p* < 0.05, ^^^^ *p* < 0.01, ^^^^^ *p* < 0.001 vs. OE‐P53; *p < 0.05, **p < 0.01, ***p < 0.001 vs. SCI; ^&^ *p* < 0.05, ^&&^ *p* < 0.01, ^&&&^ *p* < 0.001 vs. SCI + ferrostatin‐1.)

Several studies have shown that P53 can regulate the expression of ALOX15.^11^, ^27^ In order to explore the molecular regulatory relationship, in this study, we first upregulated the expression of DHODH and P53 in erastin‐treated pc12 cells in vitro to determine its regulatory role. Western blot and qRT‐PCR results showed that P53 overexpression promoted the expression of ALOX15 in pc12 cells, but the P53‐regulated ALOX15 upregulation was significantly reversed by upregulation of DHODH (Figure 5A, Figure S2A). Immunofluorescence staining also showed that ALOX15 was regulated by P53 and DHODH, and the results were statistically significant (Figure 5B,C). We further explored this specific mechanism in vivo, and as we expected, western blot and qRT‐PCR detection of ALOX15 expression in each group showed that inhibition of DHODH could upregulate the expression of ALOX15 compared with SCI group, while the ferrostatin‐1 group would effectively limit the high expression of ALOX15 (Figure 5D, Figure S2B), which was also confirmed by the results of immunofluorescence staining (Figure 5E,F). In conclusion, these results confirm that ALOX15 participates in and promotes neuronal ferroptosis and is also regulated by upstream DHODH as well as P53. These results provide the possibility for us to further discuss its mechanism.

### 3.6 DHODH inhibits neuronal ferroptosis after spinal cord injury by inhibiting P53/ALox15

**FIGURE 6:**
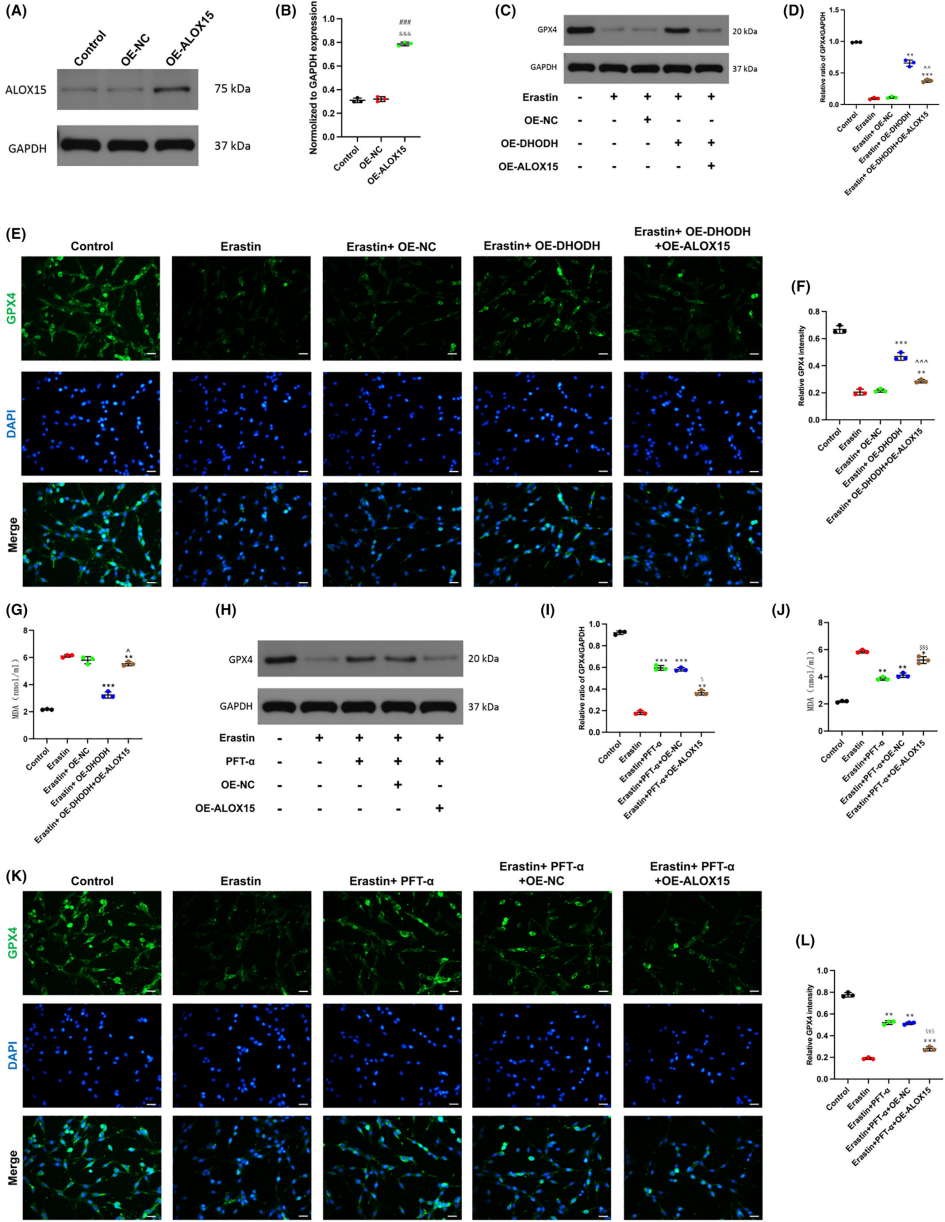
Dihydroorotate dehydrogenase inhibits neuronal ferroptosis after spinal cord injury by inhibiting P53/ALox15. (A, B) The expression of OE‐ALOX15 in transfected pc12 cells. (C, D) Western blot detection and quantification of GPX4 expression under treatment with erastin or up‐regulation of DHODH or up‐regulation of ALOX15. (E, F) The expression of GPX4 was detected by immunofluorescence staining and quantitative statistical analysis (scale bar = 50 μm, the relative content of GPX4 was calculated by Image J software). (G) MDA content in each group was detected by MDA kit. (H, I) After treatment with P53 inhibitor, the expression of GPX4 was detected by Western blot and quantitatively counted. (J) Detecting the MDA content of each group after treatment with P53 inhibitor. (K, L) Immunofluorescence staining showed that inhibition of P53 activity significantly promoted the expression of GPX4, and overexpression of ALOX15 reversed this effect (scale bar = 50 μm, the relative content of GPX4 was calculated by Image J software). (All the data are expressed as means ± SD, *n* = 3, one‐way ANOVA followed by Tukey's post hoc test was applied *p < 0.05, **p < 0.01, ***p < 0.001 vs. Erastin; ^#^ *p* < 0.05, ^##^ *p* < 0.01, ^###^ *p* < 0.001 vs. Control; ^^^ *p* < 0.05, ^^^^ *p* < 0.01, ^^^^^ *p* < 0.001 vs. Erastin + OE‐DHODH; ^&^ *p* < 0.05, ^&&^ *p* < 0.01, ^&&&^ *p* < 0.001 vs. OE‐NC; ^$^ *p* < 0.05, ^$$^ *p* < 0.01, ^$$$^ *p* < 0.001 vs. Erastin + PFT‐α.)

We hypothesized that DHODH inhibits neuronal ferroptosis after SCI by inhibiting the P53/ALOX15 pathway. Next, we further investigated upregulation of DHODH and ALOX15 to determine other regulatory roles on ferroptosis. Western blot and qRT‐PCR results indicated that overexpression of ALOX15 had acceptable efficiency (Figure 6A,B, Figure S3C). The results of erastin‐induced ferroptosis, western blot, and immunofluorescence staining showed that the expression of GPX4 increased after upregulation of DHODH expression. However, after simultaneous overexpression of ALOX15, ALOX15 effectively reversed the inhibition of DHODH on ferroptosis (Figure 6C–F). The MDA detection further confirmed the role of DHODH and ALOX15. DHODH downregulated MDA content, while upregulation of ALOX15 significantly increased MDA content (Figure 6G). For a more complete validation, we treated cells with P53 inhibitor (PFT‐α, 30 μM, 2 h) in vitro and observed the expression of GPX4. We found by western blot and immunofluorescence that inhibition of P53 activity significantly promoted the expression of GPX4, and this upregulation was effectively inhibited after overexpression of ALOX15, thereby inducing ferroptosis (Figure 6H,I,K,L). In addition, our MDA assay results also confirmed this phenomenon (Figure 6J). Based on these data, the P53/ALOX15 signaling pathway is involved in the regulation of DHODH‐inhibited neuronal ferroptosis after spinal cord injury.

## 4 DISCUSSION

Spinal cord injury (SCI) is a serious traumatic nervous system disease. After the death of spinal cord neurons, the nerve fibers of information transmission are interrupted, resulting in sensory, motor, and autonomic nerve dysfunction,^28^ which brings a great burden to the patients physically and mentally as well as social economy. Different cell death subroutines will eventually result from the damage process, which is aided by neuroinflammation, oxidative stress, lipid peroxidation, and ion imbalance.^29^ The imbalance of the microenvironment after SCI and the large number of nerve cells dying in the acute stage are important reasons why SCI is difficult to repair, so the intervention of cell death pathway is one of the main strategies for repair.^30^ The programmed cell death pathways found in SCI mainly include apoptosis, autophagy, pyropsis, necrotizing apoptosis and ferroptosis.^31^, ^32^, ^33^, ^34^ Ferroptosis is an iron‐dependent programmed mode of death newly named in 2012.^3^, ^35^ Ferroptosis is significantly different from other forms of cell‐mediated death in terms of genetic, metabolic, and protein regulatory mechanisms.^3^ After ferroptosis was proposed, some scholars found morphological, biochemical, and molecular evidence of ferroptosis in SCI during the pathological process of SCI, which is caused by ROS increase and iron accumulation in the injured site after blood spinal barrier and blood vessel rupture at the acute stage of spinal cord injury.^36^, ^37^ Therefore, preventing excessive lipid peroxidation and intracellular iron accumulation, two pathologic processes that cause ferroptosis, may be a feasible therapeutic approach for reducing spinal cord tissue injury and hastening neurological recovery. In order to successfully simulate ferroptosis in pc12 cells in vitro, erastin, a ferroptosis inducer that targets the glutamate‐cysteine anti‐transport system Xc‐, was utilized. Ferroptosis‐related chemicals and MDA were found to confirm the success of the ferroptosis model. In the in vivo study, SCI model was performed on female rats, and it was found that the expression of ferroptosis‐related molecules ACSL4 and COX2 was significantly increased in the spinal cord of rats (Figure 2), which is consistent with the current cognition that the characteristics of ferroptosis include increased Fe^2+^, accumulation of lipid ROS, upregulation of GPX4 and downregulation of ACSL4. Therefore, ferroptosis is indeed involved in the pathological process of SCI.

As a flavin‐dependent enzyme, DHODH (dihydroorotate dehydrogenase) is crucial for the de novo synthesis of pyrimidine. Dihydrocitrate is rotated by DHODH via a redox process, which results in the production of uridine monophosphate, an RNA nucleotide essential for the formation of ribosomes.^37^ DHODH expression and enzymatic activity have been implicated in cancer progression in previous studies, but as a key enzyme in pyrimidine biosynthesis, DHODH has only recently become an attractive target for anticancer therapy. At the same time, DHODH inhibitors have proven their therapeutic potential in the literature. Currently known inhibitors such as leflunomide metabolites and teriflunomide are effective therapeutic agents for the treatment of autoimmune diseases such as rheumatoid arthritis (RA).^38^ However, the mechanism of action of DHODH in various malignancies and other diseases remains unclear. Recently, Mao et al. showed that DHODH inhibits cellular ferroptosis in tumor cells. In addition, their study also found that the substrates and products of DHODH may affect the sensitivity of GPX4 to inhibit ferroptosis. Therefore, DHODH may be an important antioxidant system that inhibits ferroptosis. However, the relationship between DHODH and ferroptosis induced by spinal cord injury has not been investigated. Therefore, in our study, we upregulated the expression of DHODH in pc12 cells in vitro, and compared with other groups, and we found that overexpression of DHODH could significantly inhibit the ferroptosis of PC12 cells induced by erastin and improve the loss of cell viability. Since mitochondrial changes are special feature of ferroptosis,^39^, ^40^ and DHODH is located in mitochondria. In order to strengthen our findings, we observed mitochondrial morphology by electron microscopy, and their morphology also changed: erastin‐induced mitochondrial contraction, mitochondrial crest collapse, mitochondrial membrane potential decline, and mitochondrial membrane rupture were all reversed by upregulation of DHODH (Figure 3). In the following animal experimental study, we used DHODH inhibitor to further verify the effect of DHODH on ferroptosis after spinal cord injury (Figure 2). At the same time, both magnetic resonance imaging and spinal cord staining or motor scores showed that DHODH inhibition made spinal cord injury more severe (Figure 1). However, the ferroptosis‐1‐treated spinal cord injury group had significantly improved ferroptosis characteristics, including the expression levels of MDA and ferroptosis markers. This series of results is similar to the previous findings of Mao et al.,^8^ and our study confirmed for the first time that DHODH has the ability to inhibit neuronal ferroptosis after spinal cord injury in vitro and in vivo, thereby alleviating spinal cord injury.

Next, we explore the specific molecular mechanism of DHODH inhibiting ferroptosis. In tumor‐related studies, P53 pathway is the main pathway involved in ferroptosis.^41^, ^42^ The significance of P53 in regulating cellular metabolism and reaction to oxidative stress has been highlighted in several recent research, showing that P53 controls transcription of several genes that influence the release of cytochrome C in mitochondrial apoptosis pathways.^43^ Since P53 has many functions, such as inhibiting tumor cell survival, it is reasonable to link P53 with ferroptosis. In the past, Wei Gu's team first reported that P53 made cells sensitive to ferroptosis by inhibiting SLC7A11. The SLC7A11 promoter region contains the P53 response element, which P53 attaches to in order to suppress its expression and make tumor cells more susceptible to drugs that trigger ferroptosis, such as erastin.^44^ In contrast, few investigations on the control of P53 in ferroptosis have addressed disorders including metabolism, immunological response, neurodegeneration, and tissue ischemia/reperfusion injury. Instead, these studies have concentrated on P53's tumor‐inhibitory role. So, it would seem that P53 plays a crucial role in these disorders. Ladds et al.^10^ found that DHODH inhibitors can promote the synthesis of P53 to kill tumors. DHODH inhibitors work by activating P53‐dependent reporter genes in cells. Zhang et al.^9^ also found that DHODH inhibitors can activate P53 to improve metabolic balance. Therefore, we simulated DHODH and P53 in PC12 cells for verification in ferroptosis of spinal cord neurons, which was consistent with previous research results. Upregulation of DHODH significantly inhibited the expression level of P53, and upregulation of P53 also significantly reversed the inhibitory effect of DHODH on ferroptosis (Figure 4).

Bromfield et al.^45^ studied in mouse sperm and found that the activity of arachidonic acid 15‐lipoxygenase (ALOX15) aggravated cellular lipid peroxidation and caused ferroptosis, while Yang et al.^27^ also found that Di (2‐ethylhexyl) phthalate (DEHP) can also induce testicular ferroptosis via ALOX15. From the perspective of ferroptosis, these studies are consistent with previous beliefs that ALOX15 is a central mediator of the conversion of oxidative stress to lipid peroxidation and cell death.^46^ So, is there an upstream and downstream regulatory relationship between P53 and ALOX15, thus affecting ferroptosis? Ou et al.^11^ first discovered that P53 is associated with AlOX15 and revealed its important role in ferroptosis. Therefore, we also speculate that the expression of ALOX15 in neurons after spinal cord injury is regulated by both, and this was verified in our in vitro and in vivo experiments, which matched the previous results. It was found that ALOX15 was indeed regulated by P53 in both rats and pc12 cells, and after the addition of overexpressed DHODH, the effect of P53 on ALOX15 was reversed (Figure 5). The specific regulation mode between P53 and ALOX15 may be related to the previously reported interaction between P53 and ALOX15, which relies on SAT1 (spermidine/spermine N1‐acetyltransferase 1) as an intermediate mediator.^11^ These data suggest that DHODH may inhibit ferroptosis in neurons after spinal cord injury by regulating the expression of P53/ALOX15.

Finally, in order to further verify our hypothesis, we conducted the experiment in two parts. The first part is to evaluate the effect of upregulation of DHODH and ALOX15 on Gpx4, and the second part is to mainly evaluate the effect of upregulation of ALOX15 and adding P53 inhibitor on ferroptosis, and both parts were tested by MDA. The results are shown in Figure 6 that ALOX15 can reverse the inhibition of DHODH on ferroptosis, and the results also show that inhibition of P53 can reduce the sensitivity of ALOX15 to ferroptosis. Then combined with the previous experimental results, we can conclude that DHODH inhibits P53/ ALOX15 and thus regulates neuronal ferroptosis after spinal cord injury. DHODH, P53, and ALOX15 are three key factors associated with ferroptosis.^47^, ^48^ DHODH is an important antioxidant system that inhibits ferroptosis in mitochondria,^49^ and P53, a tumor suppressor, is essential for the prevention of tumors. In many human cancers,^50^, ^51^, ^52^, ^53^ P53 function is disrupted through mutations in the P53 gene and other mechanisms, and it has been increasingly studied in ferroptosis recently, with an increasing number of studies implicated in ferroptosis.^54^, ^55^ ALOX15 is thought to link upstream P53, DHODH, and downstream ferroptosis. According to our study, DHODH inhibited the expression of neuronal P53/ALOX15, which may be a mechanism by which DHODH mediates ferroptosis after SCI.

Although the role of DHODH is to reduce neuronal ferroptosis after SCI, the ultimate goal is to promote functional recovery after SCI. The efficacy of DHODH was confirmed by imaging, histological results, and functional behavior recovery observed in our experimental results. In addition, we also explored that DHODH inhibits ferroptosis in neurons by inhibiting the P53/ALOX15 pathway. Although the exact mechanism of action of DHODH is still unclear, we speculate that DHODH can interact with cytokines in neurons to alleviate spinal cord injury.

Recent studies have shown significant gender differences in gene expression and metabolism in the central nervous system. Chandra et al.^56^ noted that the expression of OXPHOS‐related genes is higher in females than in males, and that male and female brain microvessels differ significantly in gene expression and protein synthesis, explaining sex‐dependent differences in microcirculation during health and disease; Cikic et al.^57^ showed that the underlying basis for sex differences in brain circulation in rats seems to be estrogen. In addition, some scholars have found that compared with men, the decrease in brain perfusion in women is more significant during the aging process^58^; Some researchers conducted experiments on mice with spinal cord injury of different genders, and the results indicated that inflammatory responses to SCI are sex‐dependent at both the level of cellular recruitment and gene expression.^59^ The pathophysiology and recovery after spinal cord injury may be gender‐related. The above studies on the central nervous system injury diseases of different genders provide certain guidance for individualized treatment. But more research is needed to fully understand the mechanisms by which these genders influence the course of the disease. The reason why we chose female rats in this study is that the short and straight urethra of female rats is convenient for postoperative care of spinal cord injury surgery and reduces unnecessary death of mice.^60^ However, it is undeniable that our subsequent experiments need to take into account the differences brought by gender on body damage and recovery.

In this study, we investigated the effect of DHODH on inhibiting ferroptosis in SCI. To the best of our knowledge, our study is the first to report a link between DHODH and spinal cord injury and ferroptosis, and the results offer a promising strategy in CNS. Inhibition of ferroptosis by DHODH serves as a suitable treatment for SCI and provides a solid theoretical foundation and pharmacological recommendations for the protection of nerve injury. But the study still has some limitations. First, DHODH is a metabolism‐related enzyme in mitochondria, and it is not clear whether DHODH affects other metabolism‐related signaling pathways in the central nervous system, or whether it plays a regulatory role through metabolic pathways. Second, the regulatory relationship between P53 and ALOX15 needs to be further verified. Although previous studies have found intermediate SAT1, its exact mechanism is still unclear. Third, our animal experiments were verified by using DHODH inhibition. Considering the uncertainties of inhibitors, including off‐target effects, etc., it may be more appropriate to use gene knockout rats or transgenic rats. Fourth, in this experiment, we limited the use of female rats, ignoring the effect of gender difference on the recovery of mice after spinal cord injury, which needs to be improved in future studies.

## 5 CONCLUSION

**FIGURE 7:**
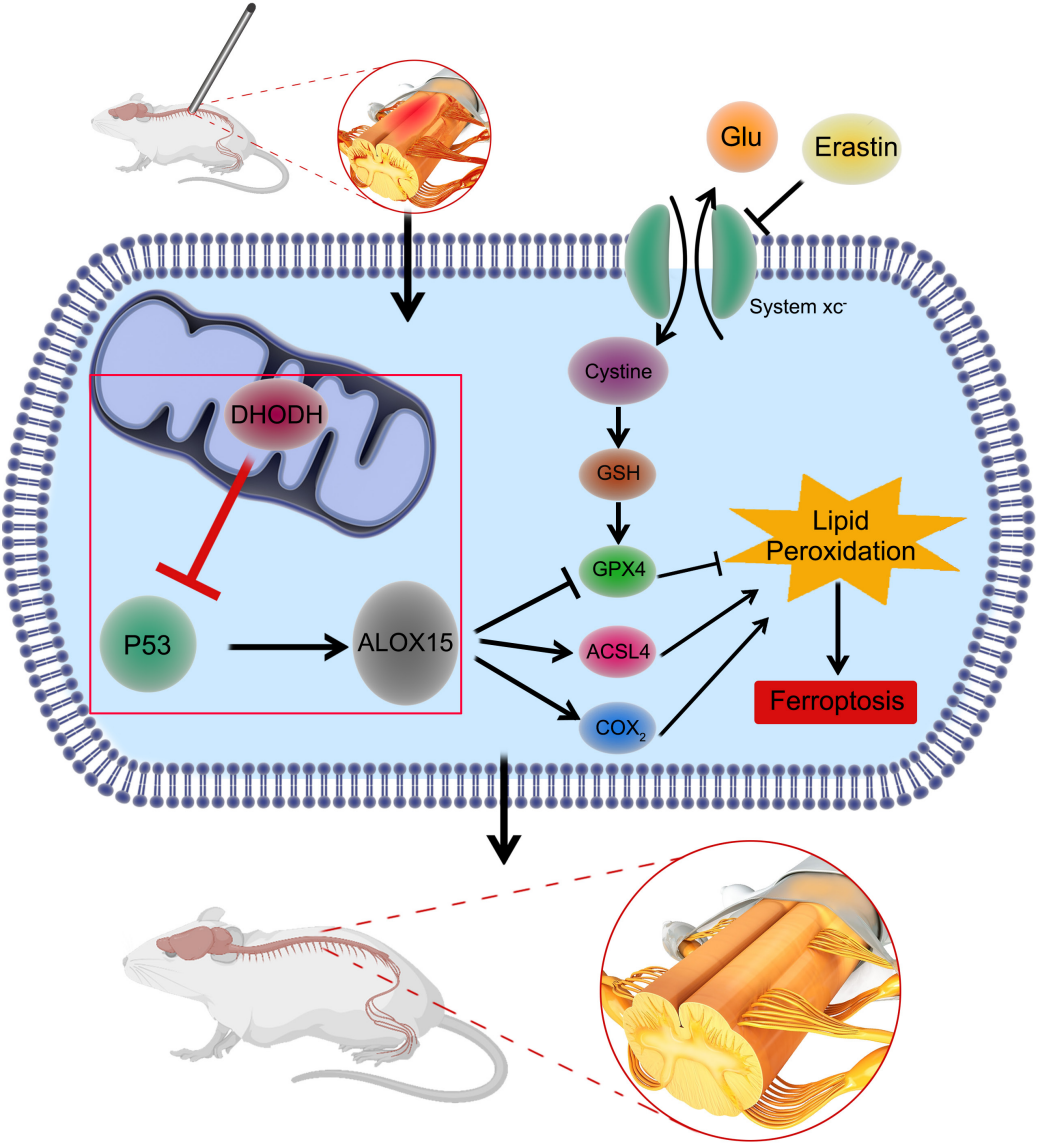
Dihydroorotate dehydrogenase regulates the ferroptosis pathway after spinal cord injury. DHODH inhibits the P53/AlOX15 signaling pathway, reduces lipid peroxidation, and ultimately inhibits ferroptosis in rat spinal cord injury models.

The application of DHOHDH is a potential treatment for SCI. DHODH can reduce the ferroptosis of neurons after spinal cord injury by regulating the P53/ALOX15 signaling pathway, thereby alleviating spinal cord injury (Figure 7).

## AUTHOR CONTRIBUTIONS

Dachuan Li, Xiao Lu, and Guangyu Xu contributed equally to this work. Hongli Wang, Fei Zou, and Xiaosheng Ma are corresponding authors. Dachuan Li, Xiao Lu, and Guangyu Xu performed the experiments and wrote the original manuscript. Siyang Liu and Zhaoyang Gong analyzed the data and prepared the figures. Feizhou Lu, Xinlei Xia, and Jianyuan Jiang have revised the manuscript. Hongli Wang, Fei Zou, and Xiaosheng Ma conceived and designed the experiments and provided funding. All authors reviewed and approved the final manuscript.

## FUNDING INFORMATION

This work was supported by the National Key Research and Development Plan, Ministry of Science and Technology of the People's Republic of China (2022YFC2407203); the National Natural Science Foundation of China, China (81972093 and 81871552); the Advanced and Appropriate Technology Promotion Project of Shanghai Municipal Health Commission, Shanghai, China (2019SY023); the Young Health Talents of Shanghai Municipal Health Commission, Shanghai, China (2022YQ011); and Hospital New Stars—Young Medical Talents (3030289002).

## CONFLICT OF INTEREST STATEMENT

Each author certifies that he or she has no commercial associations (e.g., consultancies, stock ownership, equity interest, patent/licensing arrangements, etc.) that might pose a conflict of interest in connection with the submitted article.

## Supporting information


Appendix S1

Tables S1–S5

## Data Availability

The datasets used and analyzed during the current study are available from the corresponding author on reasonable request.
