# Supplementary material for: Dihydroorotate dehydrogenase regulates ferroptosis in neurons after spinal cord injury via the P53‐ALOX15 signaling pathway
Source: CNS Neurosci Ther. 2023 Mar 21;29(7):1923–39. doi: 10.1111/cns.14150 (PMC10324365; doi:10.1111/cns.14150)
Supplement: Supplementary file 1 — Appendix S1 [file CNS-29-1923-s001.pdf]

A.

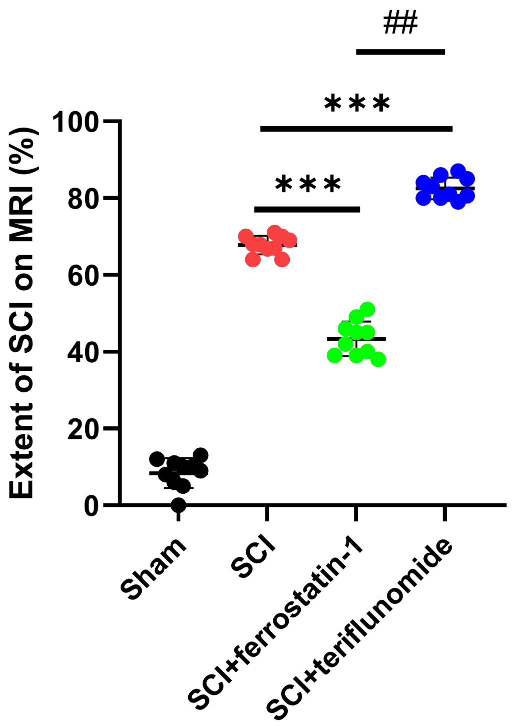

Figure S1. Quantification of animal experiment data. (A) Quantification of the extent of spinal cord injury on MRI. Proportion of spinal cord high signal within 2mm of spinal cord injury site. (All the data are expressed as means ± SD, one-way ANOVA followed by Tukey's post hoc test was applied; *P<0.05, **P<0.01, ***P<0.001 vs. SCI; #P < 0.05, ##P < 0.01, ###P < 0.001 vs. SCI+ferrostatin-1.)

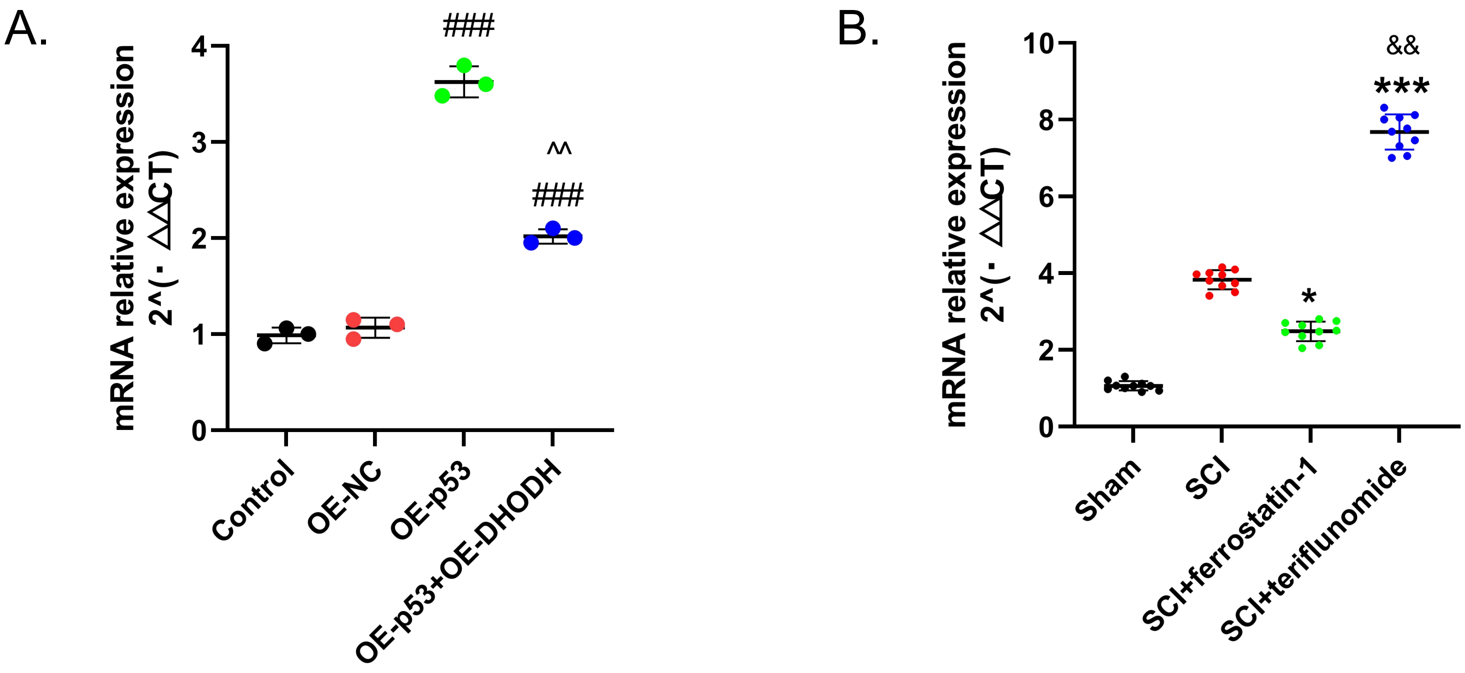

Figure S2. Quantitative results by qRT-PCR *in vitro* and *in vivo*. (A) After up-regulating the expression of p53 and DHODH, ALOX15 mRNA expression were identified by RT-PCR in PC12 cells (n = 3). (B) Relative mRNA expression of ALOX15 after SCI in rats (n = 10). (All the data are expressed as means ± SD, one-way ANOVA followed by Tukey's post hoc test was applied. $^{\#}P<0.05$, $^{\#\#}P < 0.01$, $^{\#\#\#}P < 0.001$ vs. Control; $^{\wedge}P < 0.05$, $^{\wedge\wedge}P < 0.01$, $^{\wedge\wedge\wedge}P < 0.001$, vs. OE-P53; * p<0.05, ** p<0.01, ***p<0.001 vs. SCI; $^{\&}P < 0.05$, $^{\&\&}P < 0.01$, $^{\&\&\&}P < 0.001$, vs. SCI+ferrostatin-1.)

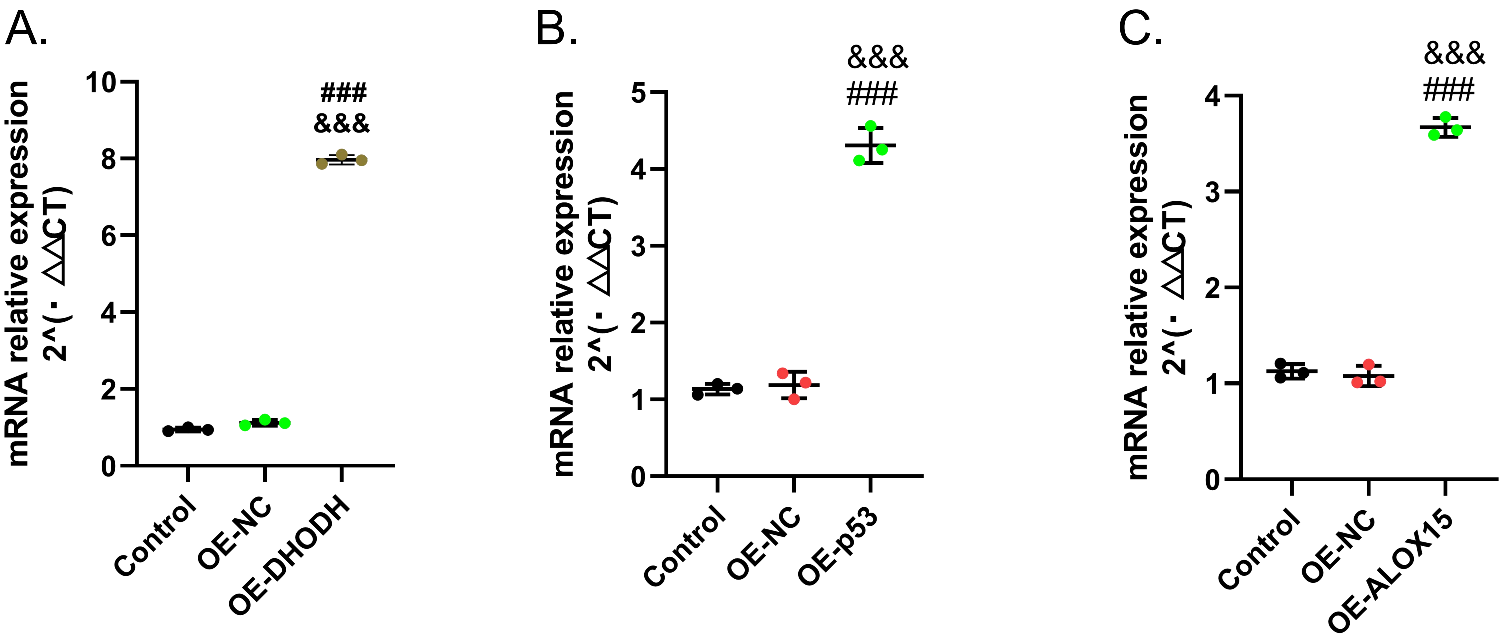

Figure S3. Quantitative results by qRT-PCR. (A) The transfection effect of overexpression of DHODH was verified at the mRNA level using qRT-PCR(n = 3). (B) The transfection effect of overexpression of p53 was verified at the mRNA level using qRT-PCR. (C)The transfection effect of overexpression of ALOX15 was verified at the mRNA level using qRT-PCR (n = 3)..(All the data are expressed as means ± SD, one-way ANOVA followed by Tukey's post hoc test was applied. $^{\#}P < 0.05$, $^{\#\#}P < 0.01$, $^{\#\#\#}P < 0.001$vs. Control; $^{\&}P < 0.05$, $^{\&\&}P < 0.01$, $^{\&\&\&}P < 0.001$vs. OE-NC.)

Target		Sequence(5′-3′)
GAPDH	Sense	AACAGCAACTCCCATT CTTCC
	Antisense	TGGTCCAGGGTTTCTT ACTCC
DHODH	Sense	GTTTCGTTGAGGTAGG AAGTGTC
	Antisense	TGTTGAATCCATACCT GTTAATGAC
P53	Sense	CGGCTCCGACTATAC CACTATC
	Antisense	GCACAAACACGAAC CTCAAAG
ALOX15	Sense	TCTACTCCACCACCT ATTTTCAC
	Antisense	TCTCTGAGATCAGGT CACTCCTG

Table S1. Primer sequence.
