# Supplementary material for: Dihydroorotate dehydrogenase regulates ferroptosis in neurons after spinal cord injury via the P53‐ALOX15 signaling pathway
Source: CNS Neurosci Ther. 2023 Mar 21;29(7):1923–39. doi: 10.1111/cns.14150 (PMC10324365; doi:10.1111/cns.14150)
Supplement: Supplementary file 2 — Tables S1–S5 [file CNS-29-1923-s002.pdf]

Figure S1

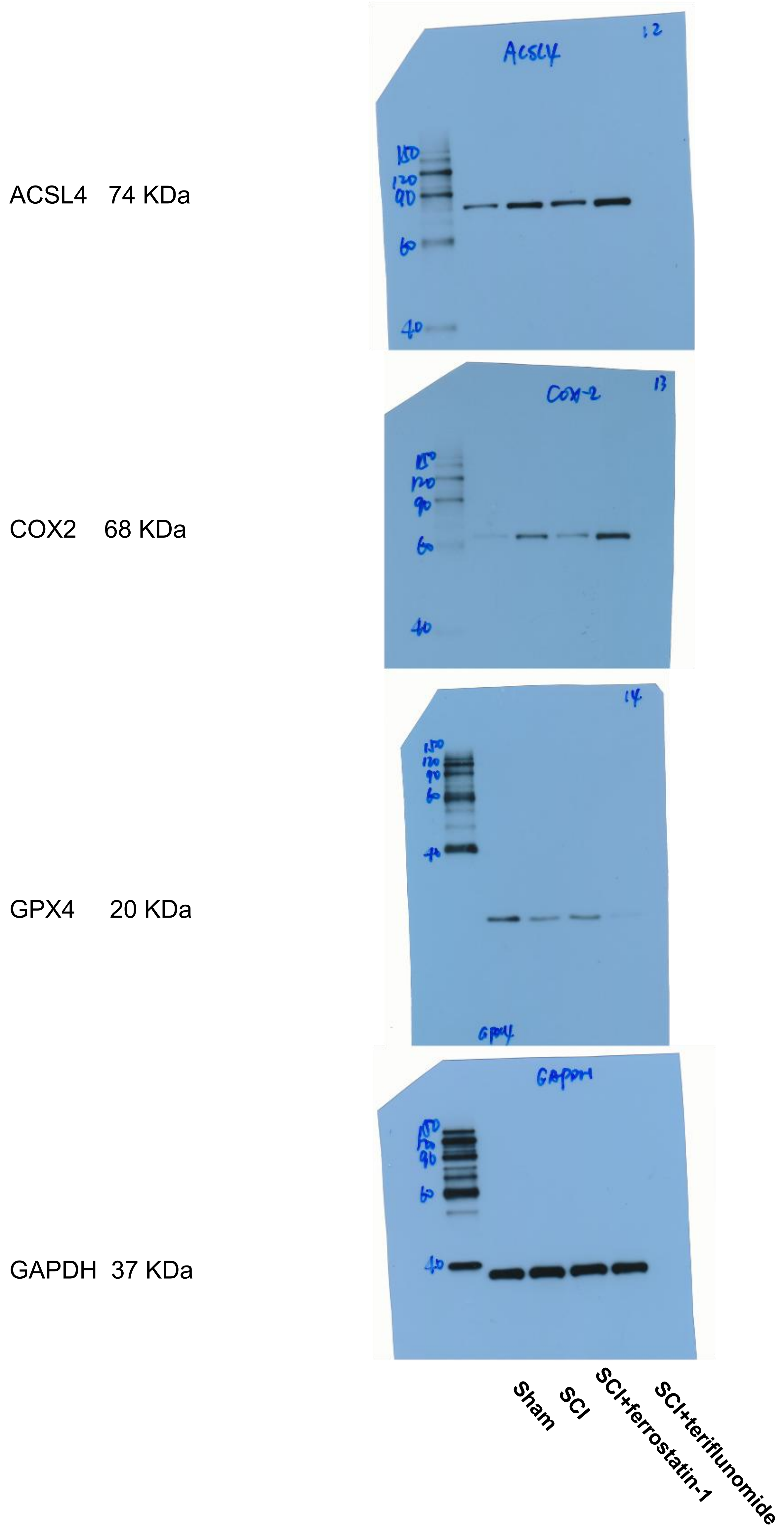

Figure S1: Full unedited gel/blot for Figure 2a.

Figure S2

(A)

DHODH 43 KDa

GAPDH 37 KDa

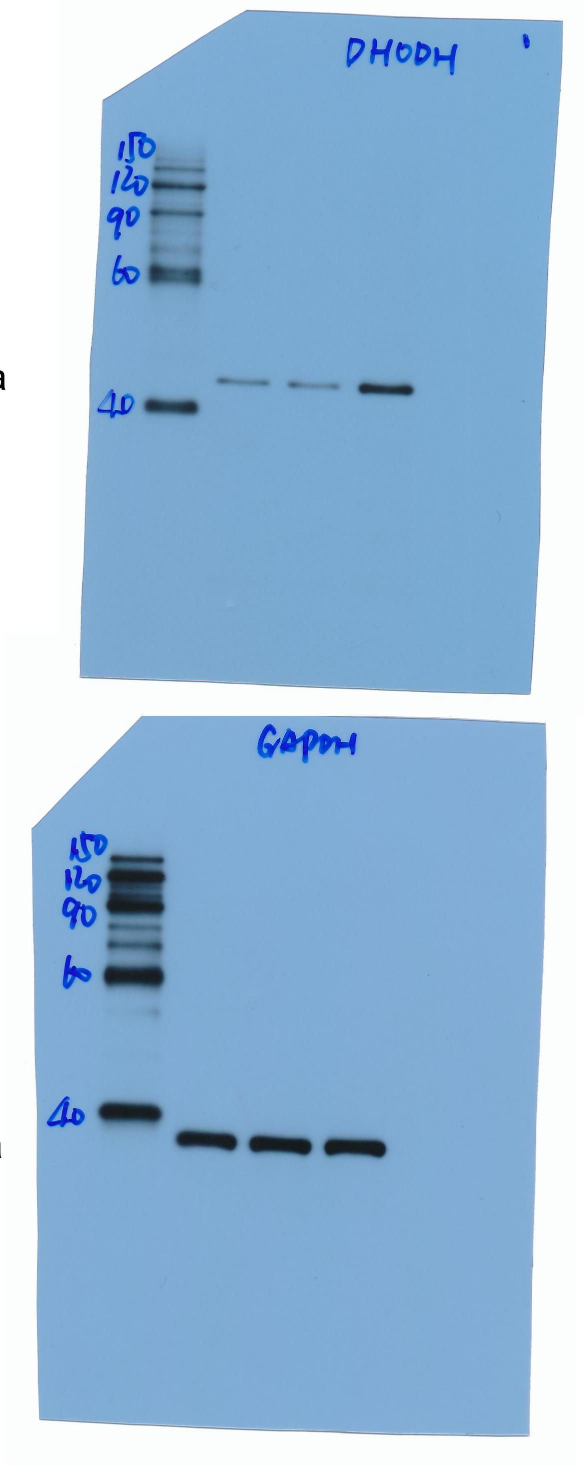

Control OE-NC OE-DHODH

(B)

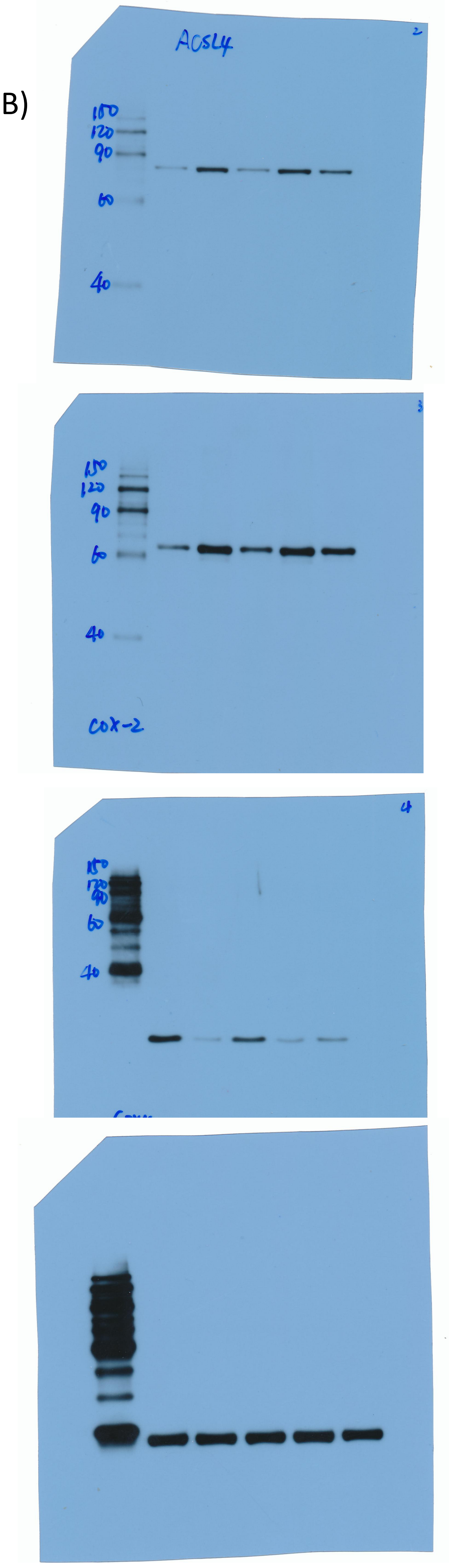

ACSL4 74 KDa

COX2 68 KDa

GPX4 20 KDa

GAPDH 37 KDa

**Erastin**	-	+	+	+	+
**ferrostatin-1**	-	-	+	-	-
**OE-NC**	-	-	-	+	-
**OE-DHODH**	-	-	-	-	+

Figure S2 (A):Full unedited gel/blot for Figure 3a.
Figure S2 (B):Full unedited gel/blot for Figure 3b.

Figure S3

(A)

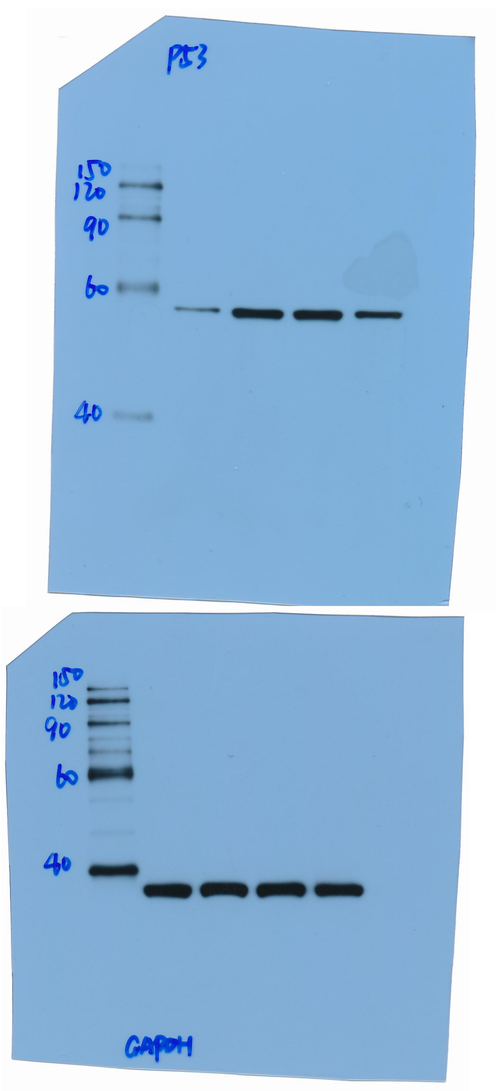

p53 53 KDa

GAPDH 37 KDa

**Erastin**	-	+	+	+
**OE-NC**	-	-	+	-
**OE-DHODH**	-	-	-	+

Figure 4

(B)

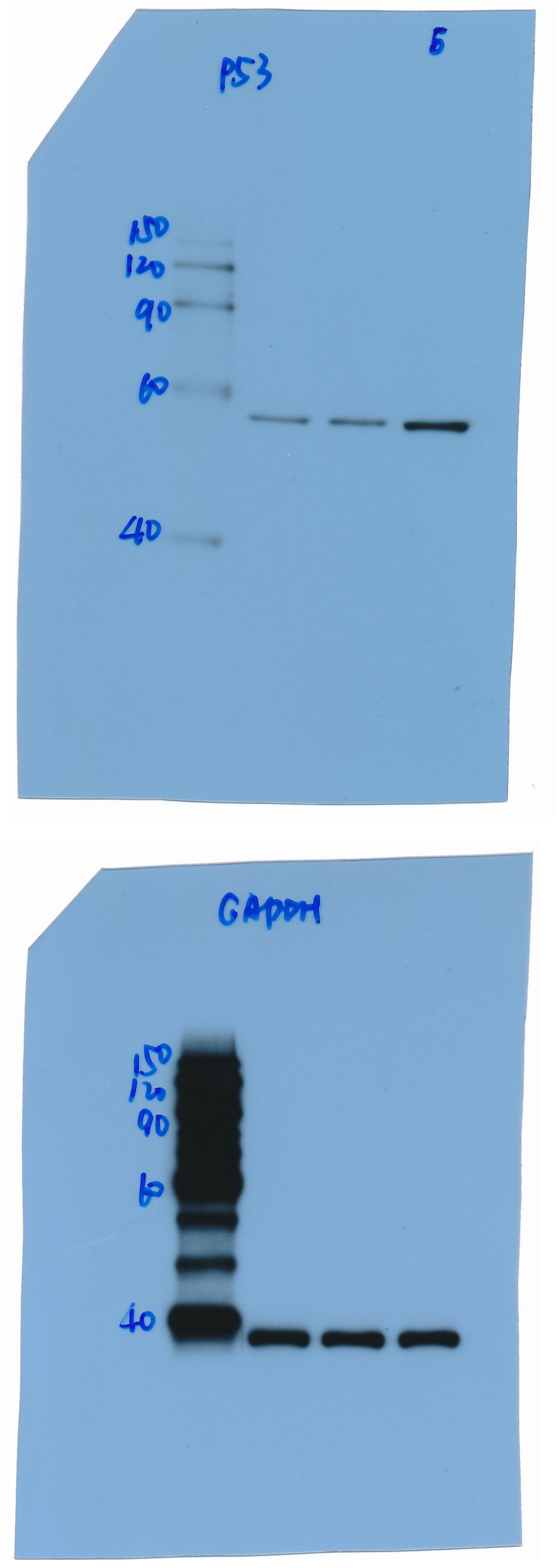

p53 53 KDa

GAPDH 37 KDa

Control OE-NC OE-p53

(C)

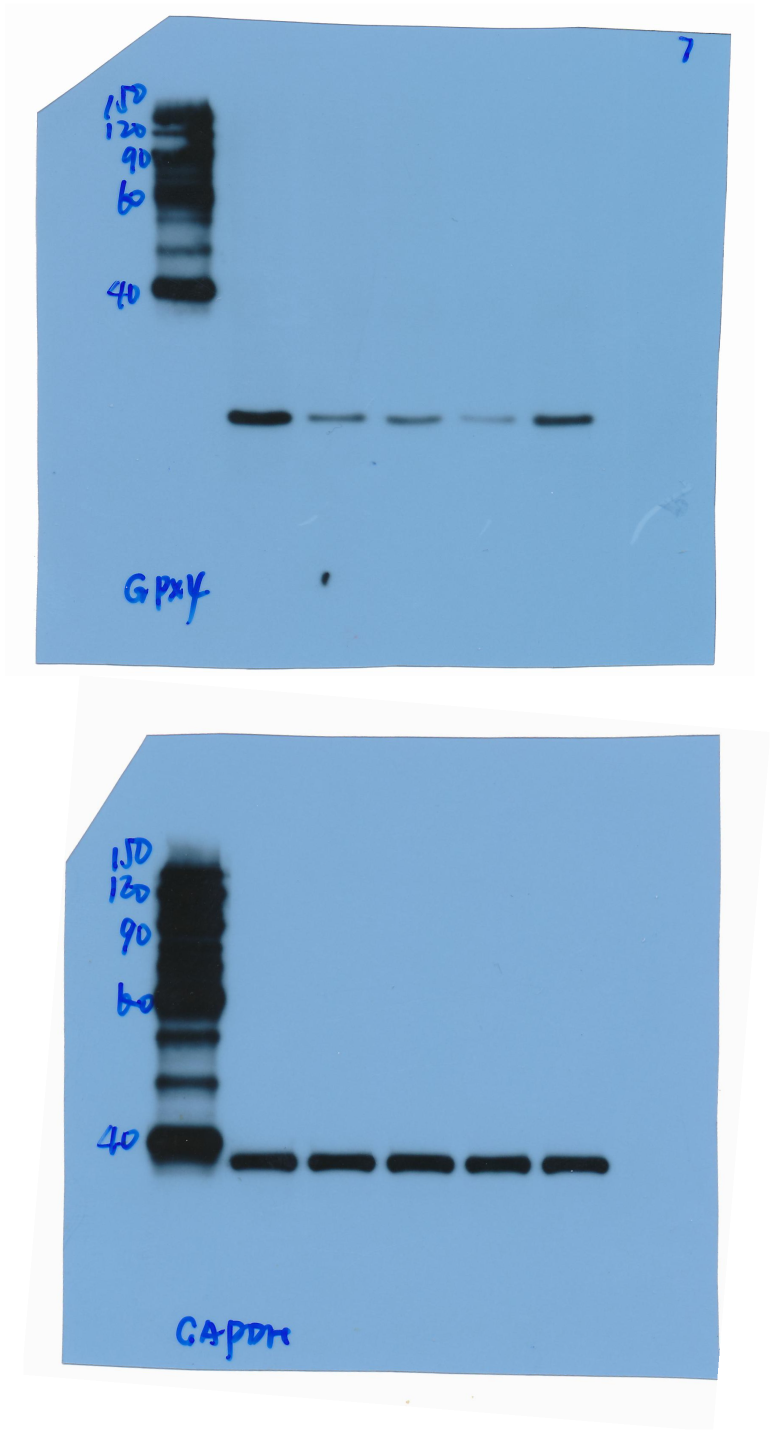

GPX4 20 KDa

GAPDH 37 KDa

**Erastin**	-	+	+	+	+
**OE-NC**	-	-	+	-	-
**OE-p53**	-	-	-	+	+
**OE-DHODH**	-	-	-	-	+

Figure S3 (A):Full unedited gel/blot for Figure 4a.
Figure S3 (B):Full unedited gel/blot for Figure 4d.
Figure S3 (C):Full unedited gel/blot for Figure 4e.

Figure S4

(A)

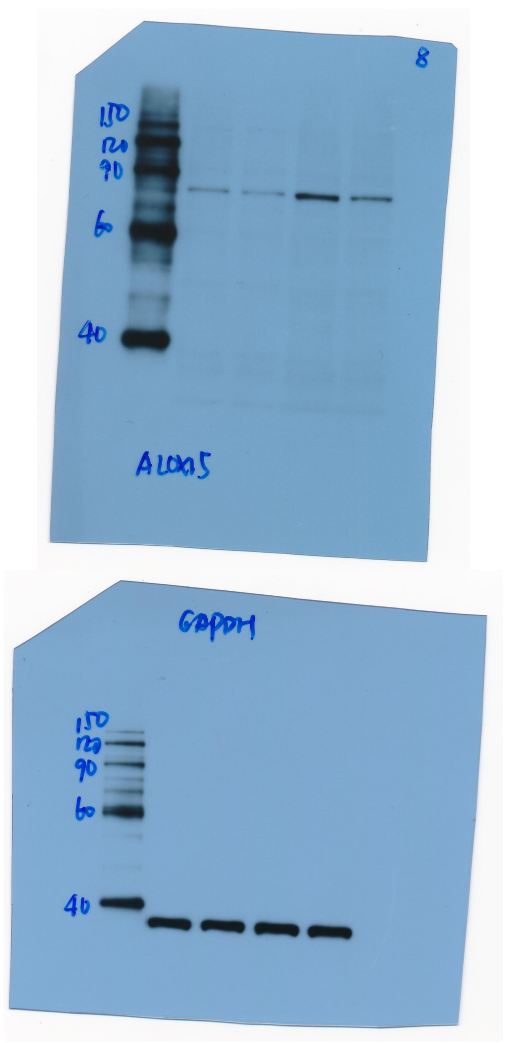

(B)

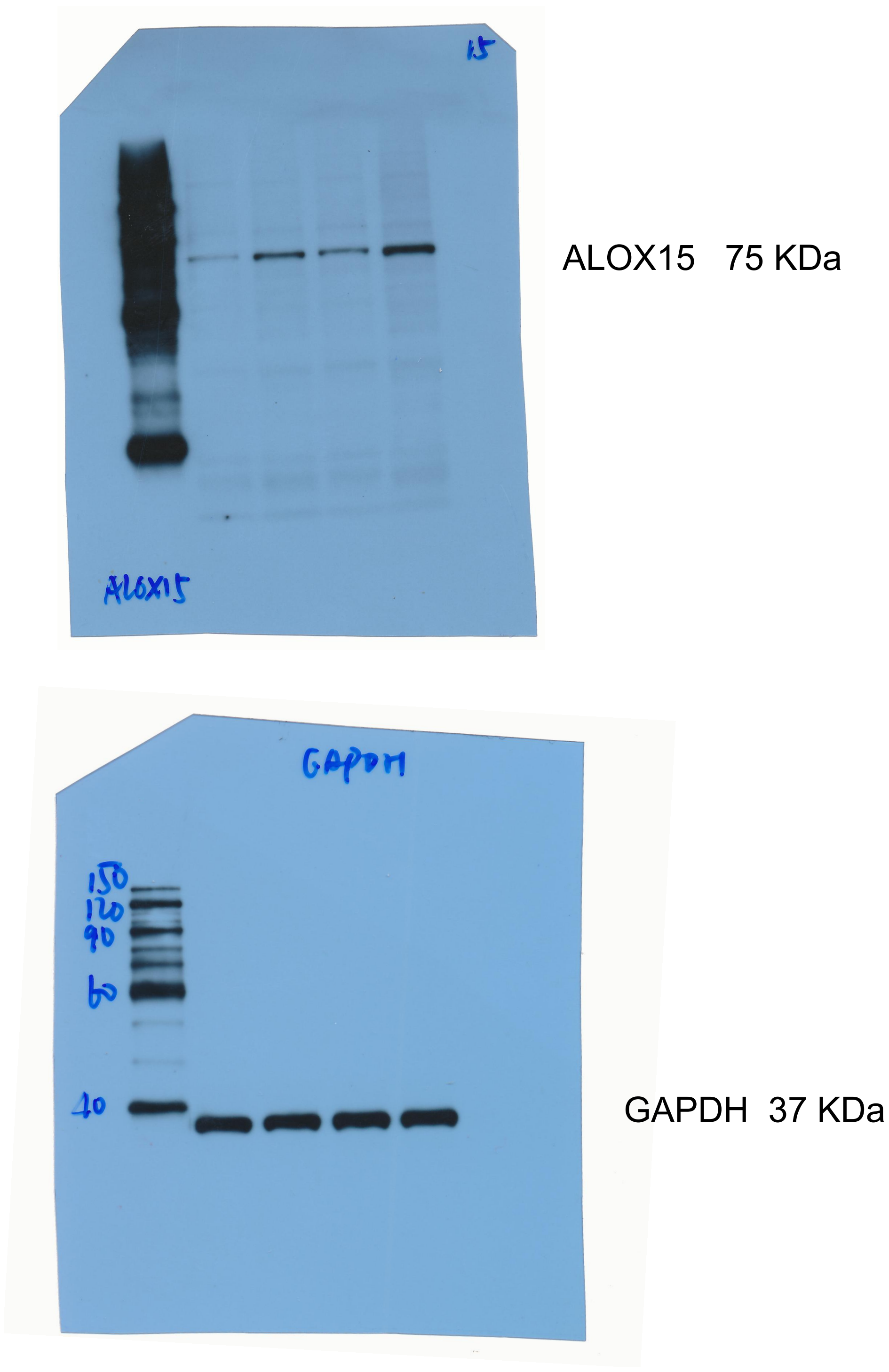

Figure S4 (A):Full unedited gel/blot for Figure 5a.
Figure S4 (B):Full unedited gel/blot for Figure 5d

Figure S5

(A)

ALOX15 75 KDa

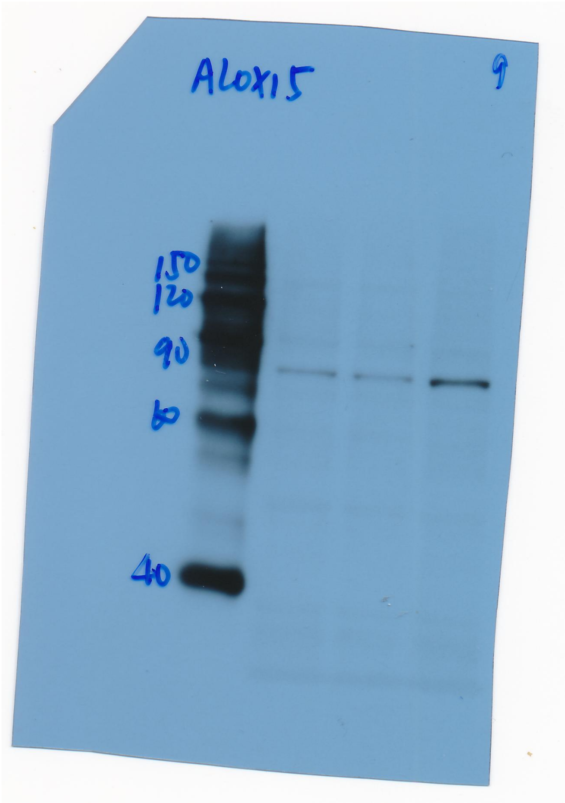

GAPDH 37 KDa

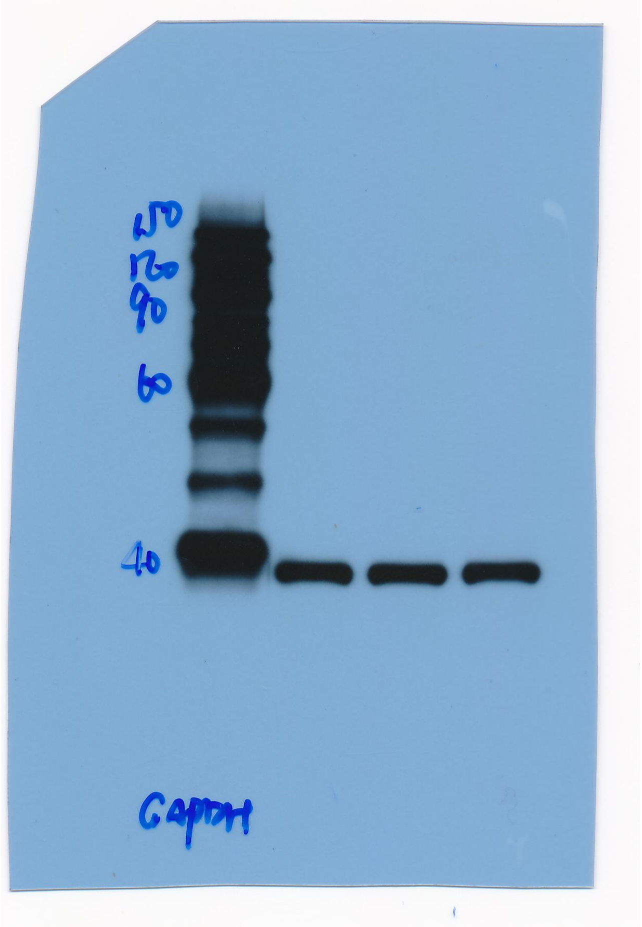

Control OE-NC OE-ALOX15

(B)

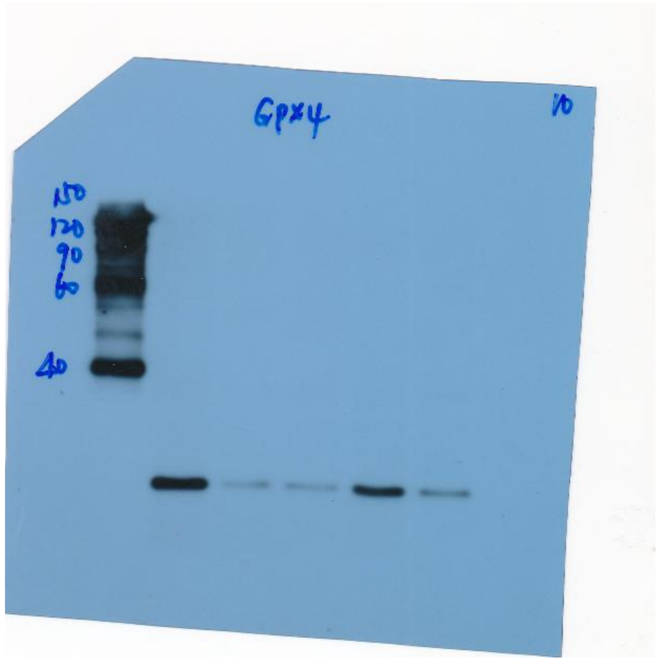

GPX4 20 KDa

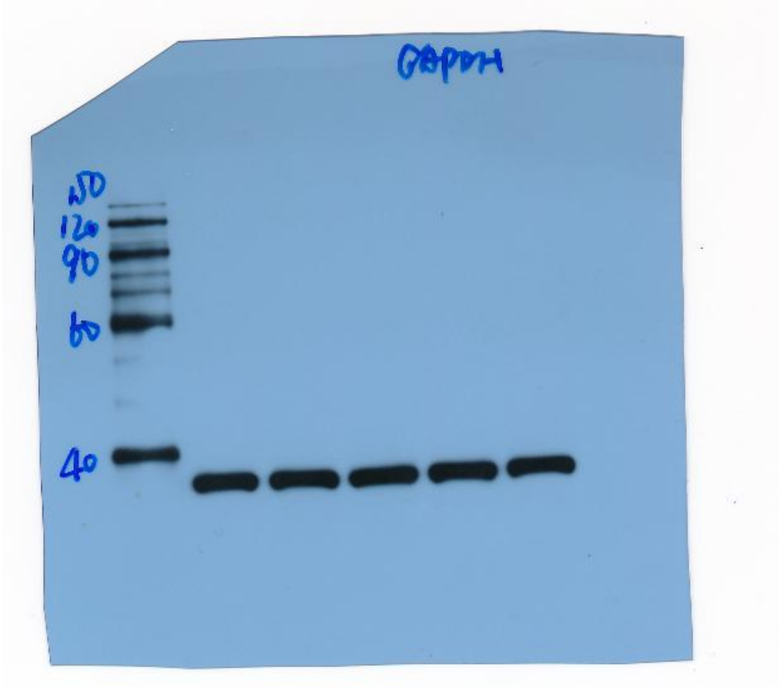

GAPDH 37 KDa

Erastin	-	+	+	+	+
OE-NC	-	-	+	-	-
OE-DHODH	-	-	-	+	+
OE-ALOX15	-	-	-	-	+

(C)

GPX4 20 KDa

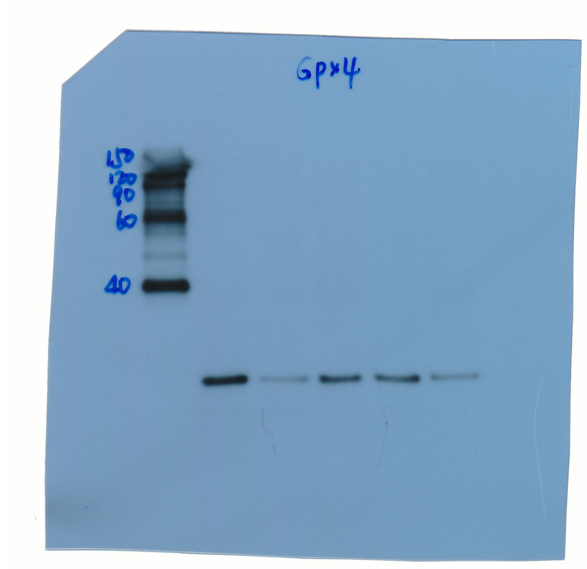

GAPDH 37 KDa

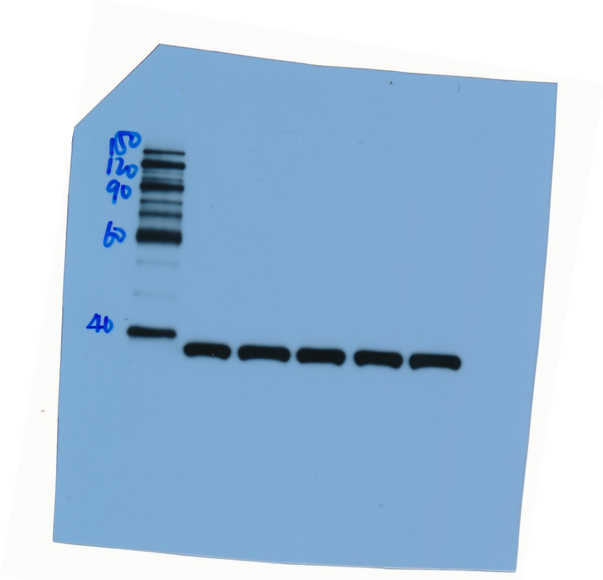

Erastin	-	+	+	+	+
PFT-α	-	-	+	+	+
OE-NC	-	-	-	+	-
OE-ALOX15	-	-	-	-	+

Figure S5 (A):Full unedited gel/blot for Figure 6a.
Figure S5 (B):Full unedited gel/blot for Figure 4c.
Figure S5 (C):Full unedited gel/blot for Figure 6h.
